# Investigational Drugs for the Treatment of Depression (Part 2): Glutamatergic, Cholinergic, Sestrin Modulators, and Other Agents

**DOI:** 10.3389/fphar.2022.884155

**Published:** 2022-06-17

**Authors:** Octavian Vasiliu

**Affiliations:** Department of Psychiatry, Dr. Carol Davila University Emergency Central Military Hospital, Bucharest, Romania

**Keywords:** treatment-resistant depression, bipolar depression, esketamine, brexanolone, glutamate, onabotulinumtoxinA

## Abstract

Many investigational drugs with antidepressant activity are currently explored in different phases of clinical research, with indications such as major depressive disorder, treatment-resistant major depression, bipolar depression, post-partum depression, and late-life depression. Although the vast majority of the antidepressants in clinical use are based on the monoaminergic hypothesis of depression, recent data supported the launching on the market of two new, non-monoamine-modulating drugs. Esketamine for treatment-resistant major depression and brexanolone for post-partum depression are two exceptions from the monoaminergic model, although their use is still limited by high costs, unique way of administration (only intravenously for brexanolone), physicians’ reluctance to prescribe new drugs, and patients’ reticence to use them. Glutamatergic neurotransmission is explored based on the positive results obtained by intranasal esketamine, with subanesthetic intravenous doses of ketamine, and D-cycloserine, traxoprodil, MK-0657, AXS-05, AVP-786, combinations of cycloserine and lurasidone, or dextromethorphan and quinidine, explored as therapeutic options for mono- or bipolar depression. Sestrin modulators, cholinergic receptor modulators, or onabotulinumtoxinA have also been investigated for potential antidepressant activity. In conclusion, there is hope for new treatments in uni- and bipolar depression, as it became clear, after almost 7 decades of monoamine-modulating antidepressants, that new pathogenetic pathways should be targeted to increase the response rate in this population.

## Introduction

Major depressive disorder (MDD) has a significant functional impact on patients’ psychosocial functioning and quality of life (Fried and Nesse, 2014). Also, individual symptoms of depression, especially sad moods and concentration problems, are associated with high levels of dysfunction in daily activities, based on an analysis of data from the STAR*D trial (Sequenced Treatment Alternatives to Relieve Depression) (Fried and Nesse, 2014). Almost 60% of individuals diagnosed with MDD report severe or very severe impairment of functioning (Kessler et al., 2003). A significant proportion of patients diagnosed with MDD will have treatment-resistant forms (TRD), which associate high direct and indirect costs, and those patients who could not reach remission have considerable healthcare resource utilization, with significant economic impact (Petrescu et al., 2014; Heerlein et al., 2022).

Patients diagnosed with bipolar disorder also may develop significant functional impairment (due to direct effects of illness severity, cognitive impairments, psychiatric comorbidities, etc.), and they spend a large duration of their lives in depressive episodes or recovering from these episodes (Levy and Manive, 2012; Solomon et al., 2016).

Postpartum depression affects up to 15% of mothers, and its short-term and long-term negative consequences on child development are well-established (Pearlstein et al., 2009). Few therapeutic options are validated for this specific pathology, and fear in mothers related to breastfeeding during antidepressant administration is a significant obstacle to efficient therapeutic management (Pearlstein et al., 2009).

Another difficult-to-treat type of mood disorder is late-life depression, where vascular factors and psychological and social factors are intertwined, and a significant risk of completed suicide is also a major threat (Vasiliu and Vasile, 2016; Alexopoulos, 2019).

New antidepressants that could be administered either as monotherapy or as an add-on to the ongoing treatment in the case of partial/inadequate response are urgently needed in clinical practice. Glutamatergic and cholinergic drugs targeting components of the hypothalamic-pituitary-adrenal axis and other non-monoaminergic systems are currently under investigation in clinical research. The main objective of this review is to explore new investigational products with antidepressant properties and their reported efficacy and tolerability in depressive disorders.

## Methodology

A systematic review of the articles referring to new drugs in phases I to III of clinical studies was conducted through the main electronic databases (PubMed, MEDLINE, Cochrane, Web of Science (Core Collection), PsychINFO, Scopus, and EMBASE using the paradigm “investigational antidepressants/products” OR “new antidepressants/agents” AND “clinical trial” AND “major depressive disorder” OR “bipolar disorder” OR “depression.” Lists of references for every article corresponding to the search paradigm were investigated, and they were added to the review if they were not detected through the previously mentioned paradigm.

A broad search was chosen to include the widest variety of molecules. For this purpose, a supplementary search was added, targeting investigational products for depression explored in the clinical trials repositories run by the United States National Library of Medicine and the National Institutes of Health (clinicaltrials.gov), World Health Organization (International Clinical Trials Registry Platform), and European Union (EU Clinical Trial Register). The search within the clinical trial databases was structured by the disorder, “depression”; type, “interventional”; population, “adults”; and “adolescents,” and trial phases I to III, but all statuses of recruitment were allowed. If the outcome of a registered trial for an investigational product was not mentioned in any of the mentioned repositories, the respective drug manufacturer’s site was explored to verify if any results were available.

All articles and references from electronic databases and clinical studies repositories included were allowed in the primary search if they were published between January 2000 and February 2022.

**FIGURE 1:**
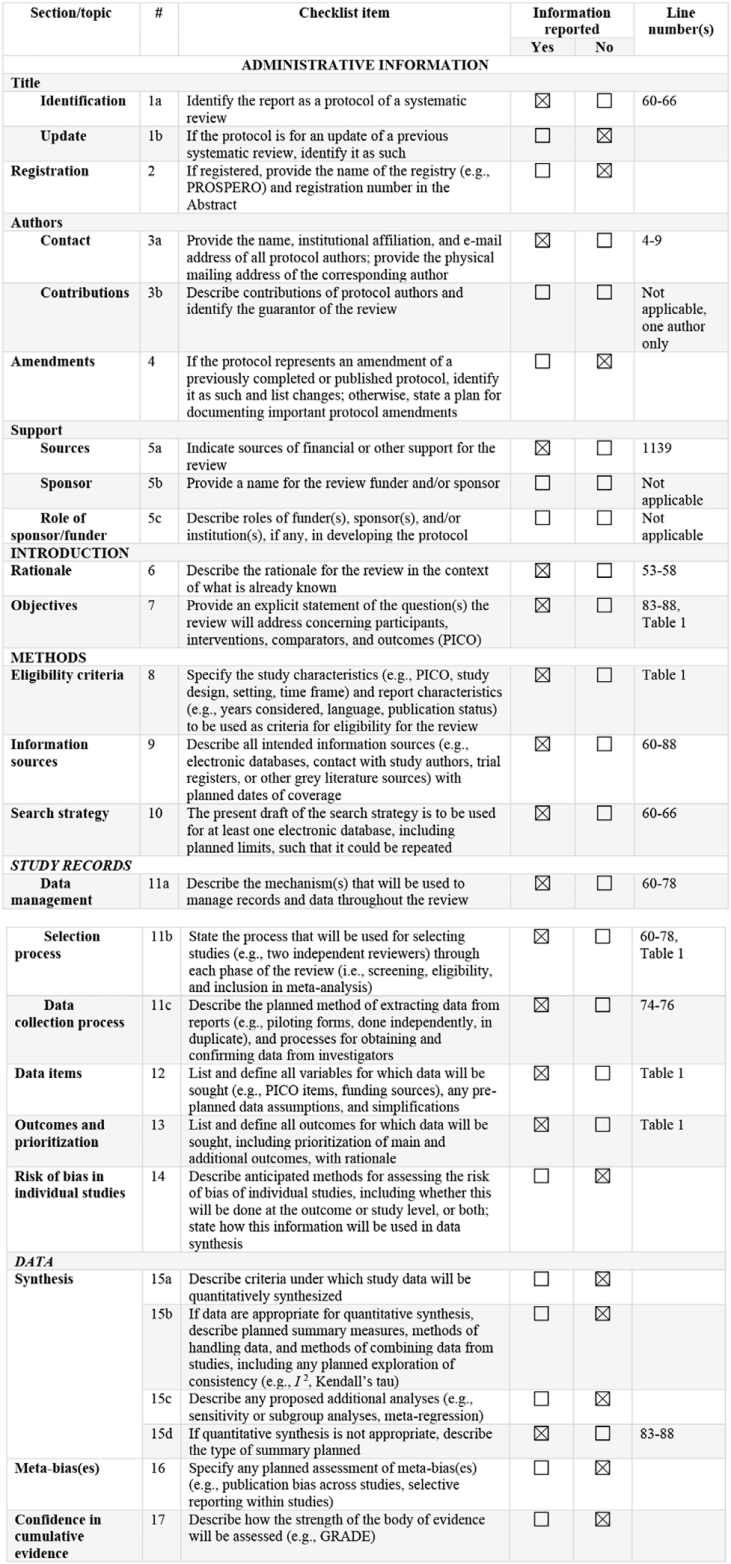
PRISMA-P 2015 Checklist (Moher et al., 2015). This checklist has been adapted for use with protocol submissions to systematic reviews from Table 3 in Moher D et al.: preferred reporting items for systematic review and meta-analysis protocols (PRISMA-P) 2015 statement. Systematic Reviews 2015 4:1.

This systematic review is based on the Preferred Reporting Items for Systematic Reviews and Meta-Analyses (PRISMA) statement, and all the data collection, review, reporting, and discussion were conducted according to this statement (Figure 1) (Moher et al., 2015). Inclusion and exclusion criteria are mentioned in Figure 4.

All pharmacological agents included in the collected data were grouped into nine categories: monoamine-based drugs, orexin receptor modulators, GABA-A receptor modulators, neurosteroid analogs, anti-inflammatory therapies, glutamatergic antidepressants, sestrin modulators, cholinergic agents, combinations of agents, and a residual category for all other molecules with distinct mechanisms of action. The first four categories of agents have been described in the first part of this review.

## Results

**FIGURE 2:**
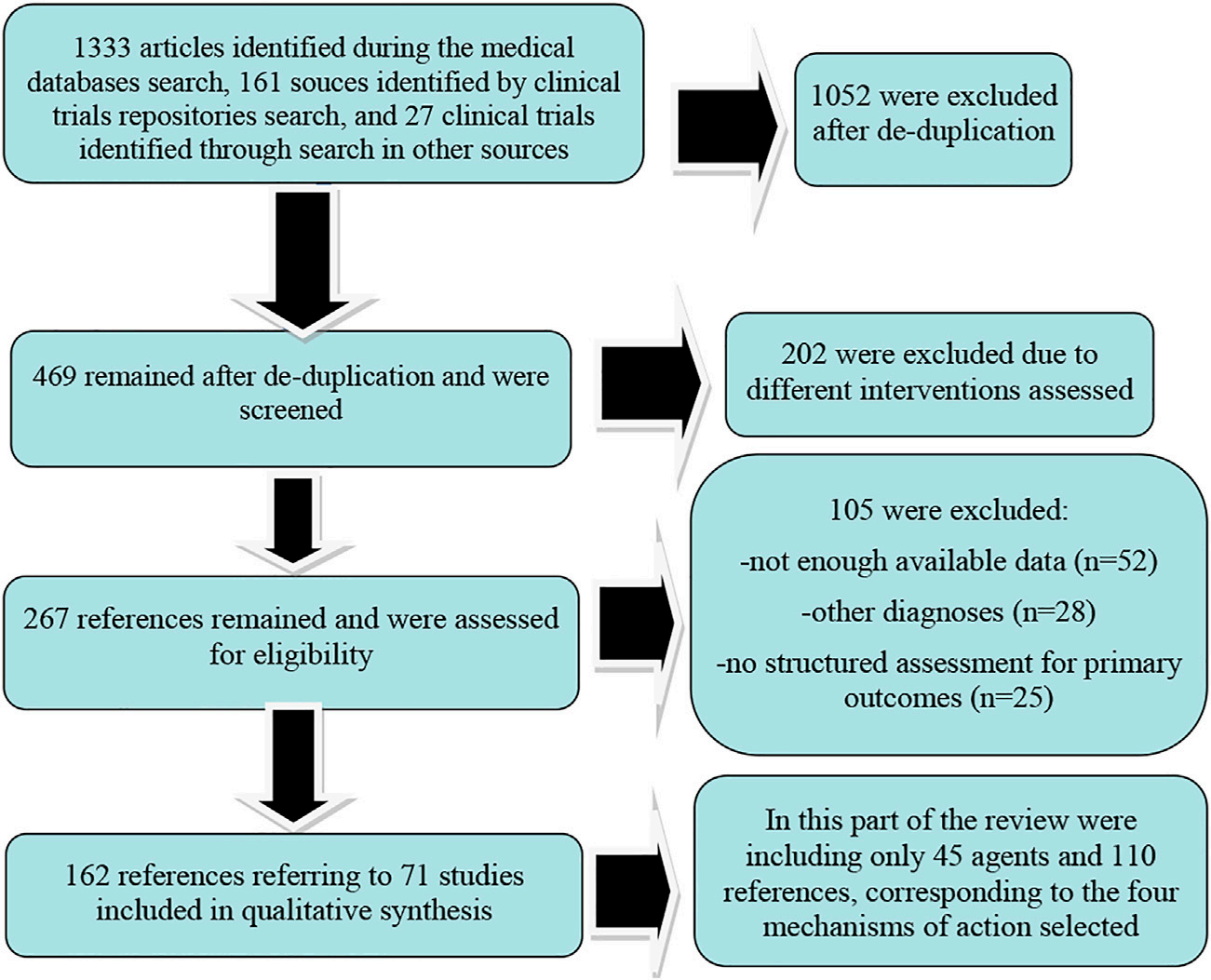
Results of the PRISMA-based search paradigm.

The results of the PRISMA-based search paradigm are presented in Figure 2. Glutamatergic agents are the most extensively researched category of antidepressants, and 29 different molecules have been found in 72 distinct sources (Table 1). Thirteen phase I studies, two phase I/II trials, 30 phase II trials, one phase II/III trial, seven phase III trials, five phase IV trials, and eight not assessed for clinical phase trials were reviewed in this category.

**TABLE 1:** Glutamatergic agents with antidepressant properties in the pipeline.

Authors	Methodology	Results	Clinical trial phase, trial identifier (if available)
GluN2B antagonists			
Preskorn et al. (2008)	Traxoprodil (CP-101,606), 6-week open-label + 4-week DBRCT, *N* = 30 MDD non-responders to open-label phase	MADRS score on day 5 (main outcome) significantly differentiated the active drug from the placebo. The response rate on HAMD was 60 *vs*. 20% for traxoprodil *vs*. placebo. The overall tolerability was good	Phase II, NCT00163059
Ibrahim et al. (2012)	Rislenemdaz (MK-0657), *N* = 5 TRD patients, 12 days	Significant antidepressant effects were reported as early as day 5 in patients receiving active drug *vs*. placebo (HAMD and BDI scores), but no improvement was observed on the MADRS (the primary efficacy measure). The tolerability was good, without dissociative AE in patients receiving MK-0657	Phase I, NCT00472576
NLM (2010a)	EVT-101, DBRCT, *N* = 8 TRD patients, 4 weeks	The primary outcome measure is the safety and tolerability profile of EVT-101. The study was prematurely discontinued due to a clinical hold issued by FDA.	Phase II, NCT01128452
NLM (2018a)	AGN-241751, two-part DBRCT, *N* = 233 MDD patients, 7 days	The primary outcome was the change in this score on day 1 and day 7 after the administration of AGN-241751. No results were posted as of February 2022	Phases I/II, NCT03726658
NLM (2018b)	AGN-241751, DBRCT, *N* = 251 MDD patients, 24 h	The efficacy at day 1 after the initial dose of AGN-241751, defined by MADRS score change, was the primary outcome. No results have yet been posted	Phase II, NCT03586427
Ghaemi et al. (2021)	Low dose/ high dose MIJ821 *vs.* placebo *vs.* ketamine, DBRCT, *N* = 70, TRD patients, 6 weeks	The adjusted mean differences *vs*. placebo were significant for all MIJ821 dosing regimens and ketamine at 24 and 48 h. At 6 weeks, none of the active interventions retained their statistical significance *vs*. placebo	Phase II, NCT03756129
NLM (2021a)	MIJ821 + comprehensive SOC, dose-ranging, *N* = 195 patients MDD + suicidal ideation/intent (estimated), 52 weeks	Primary outcome measure, MADRS total score at 24 after the first infusion and up to 52 weeks. Secondary outcomes, treatment-emergent AE (number and severity), pharmacokinetics, response rate, sustained response rate, remission rate, sustained remission rate	Phase II, NCT04722666
Fava et al. (2022)	High-dose/low-dose dextromethadone (REL-1017) adjunctive to ongoing antidepressant treatment, DBRCT, *N* = 62 TRD patients, 7 days	Patients experienced mild or moderate AE during the 7 days of the trial, with no evidence of dissociative or psychotomimetic/ opioid/withdrawal signs. MADRS scores improved on day 4 in both REL-1017 groups and persisted up to 14 days	Phase IIa, NCT03051256
NLM (2021b)	REL-1017 adjunctive to antidepressant treatment, two DBRCT, *N* = 400 MDD patients for each trial (estimated enrollment), 28 days	The primary outcome measure is MADRS total score change from baseline to day 28. These trials are ongoing as of February 2022	Phase III, NCT04688164 Phase III, NCT04855747
NLM (2021c)	REL-1017 as monotherapy, DBRCT, *N* = 400, MDD, 28 days	The primary outcome measure is MADRS total score change from baseline to day 28. The trial is ongoing	Phase III, NCT05081167
NLM (2021d)	REL-1017 as adjunctive to current antidepressant treatment, open-label, *N* = 600 MDD patients (estimated enrollment), 52 weeks	MADRS total score change from baseline to week 52 is the primary outcome. This trial is ongoing as of February 2022	Phase III, NCT04855760
Agbo et al. (2017)	AZD6765 (lanicemine), open-label and DBRCT, respectively, *N* = 46 and 40, respectively, healthy subjects, 6 days	Pharmacokinetic analysis was performed by non-linear mixed-effects modeling. The population pharmacokinetic model adequately described the clinical observation of lanicemine in healthy volunteers	Phase I, NCT01069822 Phase I, NCT00785915
Agbo et al. (2017)	AZD6765, DBRCT, single dose or multiple infusion, respectively, *N* = 34 and 152, respectively, treatment-resistant MDD patients, 24 h and 3 weeks, respectively	Pharmacokinetics parameters were already mentioned above. The overall tolerability of 100 mg lanicemine was good, and an antidepressant effect was detected after single-dose infusion, peaked at 72 h, and dissipated *vs*. placebo by 10–13 days. In the multiple-dose trial, 100 and 150 mg lanicemine were compared to placebo, and MADRS total score changed significantly at week 3 in the active drug groups. Most secondary outcomes (HAMA, QIDS-SR, Q-LES-Q) supported the significant improvement in MADRS at week 3 in the 100 mg lanicemine group	Phase IIa, NCT00491686 Phase IIb, NCT00781742
Sanacora et al. (2017)	AZD6765 (50/100 mg) adjunctive to ongoing antidepressant treatment, DBRCT, *N* = 302 MDD patients with inadequate response to treatment, 12 weeks	Lanicemine was generally well-tolerated, but neither dose was superior to placebo in decreasing the severity of the depressive symptoms (MADRS total score, QIDS-SR, SDS, CGI)	Phase IIb, NCT01482221
Zarate et al. (2013)	AZD6765 (150 mg), DBRCT, *N* = 22 TRD patients, 7 days	MADRS score significantly improved, within 80 min, in subjects receiving AZD6765 compared to placebo, and this improvement remained significant only through 110 min. The HAMD scores reflected a difference between groups at 80 and 110 min and also on day 2. The response rate was 32% in the AZD6765-treated group *vs*. 15% in placebo-treated patients. No difference between groups was reported in the rate of psychotomimetic and dissociative AE	Phase II, NCT00986479
AMPA receptor potentiators			
O’Donnell et al. (2021)	TAK-653, five escalating doses *vs*. placebo, three-crossover phases, DBRCT, *N* = 24 healthy volunteers, three phases of 1 day each, separated by wash-out periods of 10–15 days	This investigational product did not affect resting motor threshold or paired-pulse responses in humans, determined by cortical sp/ppTMS	Phase I, NCT03792672
NLM (2015a)	TAK-653, escalating single and multiple doses *vs*. placebo, *N* = 88 healthy volunteers, 14 + 31 days	The overall tolerability of the investigational product was good; no SAE were reported	Phase I, NCT02561156
NLM (2017a)	TAK-653, DBRCT, TRD patients, 57 days	The primary outcome was time to relapse (MADRS total score). The study was withdrawn (business decision). No subject has been reported as enrolled in this trial	Phase II, NCT03312894
NLM (2021e)	(2R,6R)-Hydroxy- norketamine, DBRCT, SAD, and MAD, *N* = 48 (estimates) healthy volunteers, 8 or 19 days (SAD, and MAD, respectively)	Primary outcome measures are related to safety and tolerability. The trial is ongoing as of February 2022	Phase I, NCT04711005
NMDA-receptor antagonist			
NLM (2016)	NRX-101 *vs*. lurasidone after stabilization on ketamine, DBRCT, four-arm trial, *N* = 22 BD patients + suicidal ideation/behavior, 42 days	The results support the superior efficacy of the ketamine followed by NRX-101 *vs*. ketamine followed by lurasidone treatment, as reflected by the BDM scores. No SAE were reported in any of these trial arms, and no significant difference in the rate of AE was observed between NRX-101 and lurasidone-treated groups	Phase II, NCT02974010
NLM (2018c)	NRX-101 *vs*. lurasidone after stabilization on ketamine, DBRCT, *N* = 72 (estimated) BD patients + suicidal ideation/behavior, 6 weeks	The main outcome of this trial is the improvement in the depressive symptoms between NRX-101 and lurasidone as measured by the MADRS total score. This trial is ongoing	Phase II, NCT03396068
NLM (2018d)	NRX-101 *vs*. lurasidone, *N* = 24 (estimated) moderate severity BD patients + suicidal ideation, 6 months	This trial is active. The main outcome is the improvement of depressive symptoms severity as measured by the MADRS for 6 months	Phases II/III, NCT03402152
NLM (2018e)	NRX-100 *vs*. placebo, DBRCT, *N* = 150 (estimated) BD patients + suicidal ideation/ behavior, 24 h	The primary outcome is the C-SSRS score. This trial is ongoing	Phase III, NCT03396601
Park et al. (2020)	AV-101 *vs*. placebo, cross-over DBRCT, *N* = 19 TRD patients, multiple time frames	No treatment effects were detected using linear mixed models, as determined by primary (HAMD score) or secondary (C-SSRS, response/remission rate) outcome measures. No differences for AE were reported at any time between groups	Phase II, NCT02484456
Murphy et al. (2021)	AV-101 9720/1440 mg) *vs*. placebo, cross-over DBRCT, 4/5 h	Only the high dose (1440 mg) of AV-101 in humans succeeded in engaging brain targets in humans	Phases I/II, NCT03583554
Preskorn et al. (2015)	Rapastinel (GLYX-13), single-dose, 1, 5, 10, or 30 mg/kg *vs*. placebo, DBRCT, *N* = 116 MDD patients with inadequate/partial response to antidepressants, 16 weeks	The effect of GLYX-13 was significant *vs*. placebo on day 7, but not different on day 14 on HAMD-17. Reductions in HAMD were most important for 5 and 10 mg/kg. No treatment-related SAE occurred during the study	Phase II, NCT01234558
Moskal et al. (2014), Preskorn et al. (2015)	GLYX-13 *vs*. placebo, DBRCT, *N* = 53, healthy volunteers, 4 weeks	Pharmacokinetic parameters were described after a single i.v. dose administration (0.5–2.5 mg/kg)	Phase I, NCT01014650
NLM (2012a)	GLYX-13 (5 or 10 mg/kg) *vs*. placebo, DBRCT, *N* = 369 MDD patients with inadequate/partial response to antidepressants, 16 weeks	The primary outcome measure is HAMD total score change. The study was completed, but the results are not disclosed	Phase II, NCT01684163
NLM (2019a)	GLYX-13 (225/450 mg i.v.), open-label extension, *N* = 61 MDD patients with inadequate/partial response to antidepressants, 48 months	The primary outcome was the number of participants who experienced an AE during the trial. The study was terminated by the sponsor in 32 cases, and 11 participants withdrew. Patients were rolled in NCT03668600, but this trial was also terminated (business decision)	Phase II, NCT02192099
NLM (2013)	Apimostinel (NRX-1074) *vs*. placebo, DBRCT, MAD, *N* = 100 healthy volunteers, 28 days	The primary outcome was observed and laboratory-confirmed safety. Undisclosed results	Phase I, NCT01856556
NLM (2015b)	NRX-1074 375/500/750 mg orally administered *vs*. placebo, DBRCT, MAD, *N* = 15 healthy volunteers, 28 days	The primary outcomes were related to safety and tolerability. Undisclosed results	Phase I, NCT02366364
Brooks (2015)	NRX-1074 *vs*. placebo, DBRCT, *N* = 140 MDD patients, 14 days	The primary outcome was the HAMD-17 total score change. Improvement reported after one dose of NRX-1074 infusion had an effect size of 0.88. It was also observed that 72% of the patients receiving the highest of the three tested doses demonstrated a clinically meaningful response at 24 h *vs*. 39% in the placebo group	Phase II, NCT02067793
NLM (2015c)	Ketamine i.v (single infusion) 0.1/0.25/0.5 mg/kg *vs*. midazolam 0.03 mg/kg (active placebo), DBRCT, *N* = 33 late-life TRD patients, 28 days	The rate of response (50% reduction on MADRS total score) at day 7 was 72.7% for 0.5 mg/kg ketamine i.v. *vs*. 46.2% for midazolam (active placebo) and 87.5 *vs*. 66.7% at day 28	Phase III, NCT02556606
NLM (2017b)	Ketamine 0.5 mg/kg *vs*. placebo, DBRCT, *N* = 64 prenatal depression patients, 48 h	EPDS score at 48 h after delivery is the main outcome measure. Undisclosed results	Phase IV, NCT03336541
Lapidus et al. (2014)	Intranasal ketamine (SLS-002) *vs*. placebo, cross-over DBRCT, *N* = 20 TRD patients, 24 h	Patients treated with SLS-002 significantly improved their depressive symptoms 24 h after drug administration *vs*. placebo (MADRS total score change), and the overall tolerability was good, with minimal AE. Response criteria were met by 8 out of the 18 patients treated with ketamine 24 h after drug administration *vs*. 1 out of 8 patients on placebo	Phase II, NCT01304147
NLM (2020), PRNewswire (2021a)	SLS-002 + SOC, *N* = 236 (estimated) MDD patients with imminent risk of suicide, two phases: the first phase is open-label, while the second is double-blind, 24 h and 16 days, respectively	Analysis of the first 17 patients enrolled in this trial demonstrated a rapid onset of antidepressant action from the first dose. Mean MADRS scores met the remission criteria on day 6. The trial is ongoing	Phase II, NCT04669665
Leal et al. (2021)	R-Ketamine (PCN-101), open-label, pilot trial, *N* = 7 TRD patients, 24 h	The mean MADRS score changed significantly, with 20.3 points in 24 h, and no clear dissociative symptoms were reported	Phase N/A
PRNewswire (2021b)	PCN-101 *vs*. placebo, DBRCT, *N* = 58 healthy volunteers	PCN-101 was safe and well-tolerated at all doses up to 150 mg, and no SAE were reported, according to the manufacturer’s press release. In the second stage of the study, the relative safety and tolerability of PCN-101 were compared to that of S-ketamine, and the results demonstrated that PCN-101 required a substantially higher dose to obtain similar perceptual changes to S-ketamine	Phase I, ACTRN12620000226909
Huang et al. (2013), NLM (2009a)	N-Methylglycine (sarcosine) *vs*. citalopram, DBRCT, *N* = 40 MDD patients, 6 weeks	Sarcosine significantly improved HAMD, CGI, and GAF scores more than citalopram treatment. Sarcosine was associated with a higher probability of symptom remission, quicker response, and less risk for dropout. The overall tolerability of sarcosine was good, without significant AE	Phase II, NCT00977353
NLM (2021f)	Sarcosine *vs*. placebo as add-on to SSRI, *N* = 60 MDD patients, 8 weeks	The primary outcome measure is the change in the severity of depressive symptoms from baseline (MADRS total score change). The trial is ongoing	Phase IV, NCT04975100
Heresco-Levy et al. (2006)	D-Cycloserine *vs*. placebo as add-on to ongoing antidepressant, cross-over DBRCT, *N* = 22 TRD patients, 6 weeks	D-Cycloserine induced symptoms reduction and was well tolerated, but the efficacy did not reach statistically significant levels in patients with D-cycloserine *vs*. placebo adjuvant treatment	Phase I
Heresco-Levy et al. (2013)	D-Cycloserine *vs*. placebo as add-on to ongoing antidepressant, DBRCT, *N* = 26 TRD patients, 6 weeks	D-Cycloserine was well tolerated, had no psychotomimetic effects, and improved depressive symptoms, as measured by HAMD and BDI at a significantly level *vs*. placebo	Phase II, NCT00408031
Chen et al. (2019)	D-Cycloserine, *N* = 32 MDD or BD patients who responded to ketamine i.v. in an open-label first phase, DBRCT, 6 weeks	Final total HAMD scores did not differ between the two groups, but a potential effect of D-cycloserine over suicide ideation/behavior was identified by mixed model analysis throughout the follow-up period	Phase II
NLM (2018f)	D-Cycloserine *vs*. modafinil + CBT, DBRCT, *N* = 36 MDD patients, 3 weeks	The primary outcome measures were the recall of CBT content, the delayed recall of emotional story items, and the delayed recall of logical memory after 2 and 3 weeks. The results have not yet been published	Phase II, NCT02376257
Chen et al. (2014)	Dextromethorphan/ placebo + valproic acid, DBRCT, *N* = 309 BD patients, 12 weeks	Plasma cytokine levels declined in all groups, and changes in BDNF levels were significantly higher in the valproic acid + dextromethorphan 60 mg/day group than in the valproic acid + placebo group	Phase N/A
Nagele et al. (2015)	Nitrous oxide *vs*. placebo, cross-over DBRCT, *N* = 21 TRD and non-TRD patients, 24 h	Depressive symptoms improved significantly at 2 and 24 h after nitrous oxide administration *vs*. placebo (according to HAMD-21 scores). Treatment response was observed in four patients (20%), and three patients had a full remission after nitrous oxide *vs*. one patient (5%) and none after placebo. No SAE occurred, and all AE were brief and of mild-to-moderate severity	Phase II, NCT02139540
NLM (2017c)	Nitrous oxide *vs*. placebo, DBRCT, *N* = 34, 24 h	The primary outcome is HAMD-21 scores at 2 and 24 h after treatment. Undisclosed results	Phase II, NCT03283670
Zarate et al. (2004)	Riluzole 168.8 mg/day, open-label trial, *N* = 19 TRD patients, 6 weeks	Significant improvement in MADRS scores occurred in weeks 3–6, in trial completers, and CGI-S and HAMA also improved significantly during weeks 3–6. The most common adverse events during the trial were headache, gastrointestinal distress, tension, or inner unrest	Phase N/A
Brennan et al. (2010)	Riluzole 100–200 mg/day, open-label trial, *N* = 14 BD patients, 6 weeks	Riluzole led to a significant reduction of HAMD scores, while the glutamine/glutamate (Gln/Glu) ratios increased significantly by day 2 of the treatment	Phase N/A, NCT00544544
Sanacora et al. (2007)	Riluzole 100 mg/day + ongoing antidepressant, open-label trial, *N* = 10 TRD patients, 6 + 6 weeks	HAMD and HAMA scores declined significantly following the initiation of riluzole augmentation treatment, and the effect of riluzole became significant at the end of the first week of the trial and persisted for the 12-week duration of monitoring	Phase N/A
NLM (2010b)	Riluzole/placebo + ongoing SSRI/SNRI, DBRCT, *N* = 104 TRD patients, three-phase study (24 weeks, in total)	Rough, unpublished data did not support a large difference between groups in the MADRS scores, while the response rate at week 8 (secondary outcome) was higher for placebo than for any of the active groups	Phase II, NCT01204918
NLM (2012b)	Riluzole + sertraline *vs*. placebo + sertraline, DBRCT, *N* = 21 MDD outpatients, 8 weeks	The primary outcome measures were the mean change in HAMD scores from baseline to endpoint and the number of patients with antidepressant response or remission at week 8. This study was prematurely terminated due to administrative reasons	Phase II, NCT01703039
NLM (2001)	Riluzole 50–200 mg/day, single-arm, single-blind, *N* = 31 MDD patients, 6 weeks	No results were posted or published	Phase II, NCT00026052
Mathew et al. (2010)	Lamotrigine *vs*. placebo pre-treatment, followed by ketamine infusion, responders were randomized on riluzole 100–200 mg/day or placebo, DBRCT; *N* = 26 recurrent or chronic MDD, 24–72 h after i.v. ketamine	An interim analysis did not find any significant differences between riluzole and placebo regarding the main outcome (time-to-relapse). The trial was discontinued for futility	Phase IV, NCT00419003
NLM (2006a)	Riluzole 100–200 mg/day *vs*. placebo, DBRCT, *N* = 94 BD patients, 8 weeks	The main outcome measure was the mean change in the MADRS score. The rough, unpublished results did not support the superior efficacy of riluzole *vs*. placebo	Phase II, NCT00376220
Zarate et al. (2005)	Riluzole 50–200 mg/day + lithium, open-label study, *N* = 14 BD patients, 8 weeks	The linear mixed model for total MADRS score showed a significant treatment effect at week 8, without cases of switch into hypomania or mania	Phase N/A
NLM (2003)	Riluzole 50–200 mg/day *vs*. placebo, DBRCT, *N* = 19 BD patients, 8 weeks	The study was terminated due to the superior efficacy of placebo in an interim analysis	Phase II, NCT00054704
Zarate et al. (2006)	Memantine 5–20 mg/day *vs*. placebo, DBRCT, *N* = 32 MDD patients, 8 weeks	The results of this trial (MADRS scores change from baseline to week 8) were negative	Phase N/A
Smith et al. (2013)	Memantine 5–20 mg/day *vs*. placebo + antidepressant, DBRCT, *N* = 31 patients with partial or non-responsive MDD, 8 weeks	No statistical differences were observed between groups on primary or secondary efficacy outcomes or safety outcomes	Phase N/A
NLM (2006b)	Memantine 5–20 mg/day + lamotrigine, DBRCT, *N* = 29 BD patients, 8 weeks	The primary outcome was the change in HAMD-17 from baseline to week 8. Unpublished results show a decrease of 9 *vs*. 7 points in patients treated with memantine *vs*. placebo. The most frequently reported adverse events in the memantine group were somnolence, indigestion, diarrhea, headache, and coughing	Phase IV, NCT00305578
NLM (2002)	Memantine 5–20 mg/day *vs*. placebo, DBRCT, *N* = 112 MDD outpatients, three-phase study (2, 8, and 16 weeks)	No results of this trial have been released	Phase III, NCT00040261
NLM (2006c)	Memantine 5–20 mg *vs*. placebo as add-on to antidepressants, DBRCT, *N* = 31 MDD patients with incomplete response/ non-response to antidepressants	The main outcome was the change in MADRS scores at week 8. Unpublished results did not support a significant difference between groups (−7.13 *vs*. −7.25 points in memantine *vs*. placebo). The rate of serious adverse events was similar in the two groups	Phase IV, NCT00344682
Metabotropic glutamate receptors antagonists			
Watanabe et al. (2021)	TP0473292 (TS-161) *vs*. placebo, DBRCT, SAD, and MAD, *N* = 70 healthy subjects, 10 days	The investigational product penetrated the brain–blood barrier, and the most frequently reported AE were nausea, vomiting, and dizziness, with an exposure-related incidence	Phase I, NCT03919409
NLM (2021g)	TS-161 *vs*. placebo, DBRCT, *N* = 25 (estimated), TRD patients, 21 days	The main outcome is the change from baseline to day 21 on MADRS total scores. The trial is ongoing	Phase II, NCT04821271
Umbricht et al. (2020)	Decoglurant (RO4995819) *vs*. placebo, DBRCT, *N* = 357 TRD patients, 6 weeks	At week 6, no significant differences were observed between any active treatment arms and placebo in decreasing MADRS scores, response, or remission rates. No effects of decoglurant were observed on CANTAB. A high rate of placebo response was observed	Phase II, NCT01457677
NLM (2012c)	RO4995819 *vs*. placebo as adjunctive therapy, DBRCT, TRD patients, 6 weeks	The main outcome measure was MADRS total score change. The trial was withdrawn by the sponsor. No subject was enrolled	Phase II, NCT01733654
Quiroz et al. (2016)	Basimglurant (RG-7090) *vs*. placebo as an adjunctive agent to SSRI/SNRI, DBRCT, *N* = 333 MDD patients, 6 weeks	No difference was observed in the primary outcome, MADRS change from baseline to the endpoint, between basimglurant MR and placebo. Secondary endpoints were modified by adjunctive basimglurant MR 1.5 mg daily, especially in patient-rated measures. The most frequently reported AE was dizziness, but it was of mild intensity and transient	Phase IIb, NCT01437657
NLM (2015d)	RG-7090 *vs*. placebo, DBRCT, MAD, *N* = 56 healthy subjects + MDD patients, 10 weeks	The primary outcomes were tolerability and safety of the investigational product. The results of this trial are undisclosed as of February 2022	Phase I, NCT02433093
NLM (2010c)	AZD-2066 *vs*. placebo *vs*. duloxetine, DBRCT, *N* = 131 MDD patients, 6 weeks	The primary outcome was MADRS totals core change from baseline to week 6. The improvement was −13.1 (AZD 2066), −14 (duloxetine), and −14.1 (placebo). The response rate was the same in all three groups	Phase IIa, NCT01145755

AE, adverse events; AMPA, alpha-amino-3-hydroxy-5-methyl-4-isoxazole propionic acid; BD, bipolar depression; BDI, Beck Depression Inventory; BDM, Bipolar Inventory of Symptoms Scale-derived MADRS; BDNF, brain-derived neurotrophic factor; CANTAB, Cambridge Neuropsychological Test Automated Battery; CBT, cognitive-behavioral therapy; CGI, Clinical Global Impression; C-SSRS, Columbia Suicidality Severity Scale; DBRCT, double-blind randomized controlled trial; FDA, Food and Drug Administration; GAF, Global Assessment of Functioning; HAMD, Hamilton Depression Rating Scale; MAD, multiple ascending dose; MADRS, Montgomery Asberg Depression Rating Scale; MDD, major depressive disorder; N/A, not applicable; NIMH, National Institute of Mental Health; NMDA, N-methyl-D-aspartate; QIDS-SR, Quick Inventory of Depressive Symptomatology-Self-Report; Q-LES-Q, Quality of Life Enjoyment and Satisfaction Questionnaire-Short Form; SAD, single ascending dose; SAE, severe adverse events; SDS, Sheehan Disability Score; SNRI, serotonin and norepinephrine reuptake inhibitor; SOC, standard of care; SSRI, selective serotonin reuptake inhibitors; sp/ppTMS, single-pulse/paired-pulse transcranial magnetic stimulation; TRD, treatment-resistant MDD.

Sestrin modulators were identified in two sources referring to one phase I and one phase II trials, assessing a single agent from this category. Four different combinations of pharmacological agents were identified in 13 sources, referring to 5 phase II trials, 8 phase III trials, and one not assessed for a clinical phase trial.

Cholinergic antidepressants have been identified in 10 distinct sources, referring to three investigational products, explored in two phase I trials, four phase II trials, two phase IV trials, and two not assessed for clinical phase trials. Eight other antidepressants with distinct mechanisms of action have been identified in 13 sources, referring to one phase I trial, seven phase II trials, two phase IV trials, and three not assessed for clinical phase trials.

**FIGURE 3:**
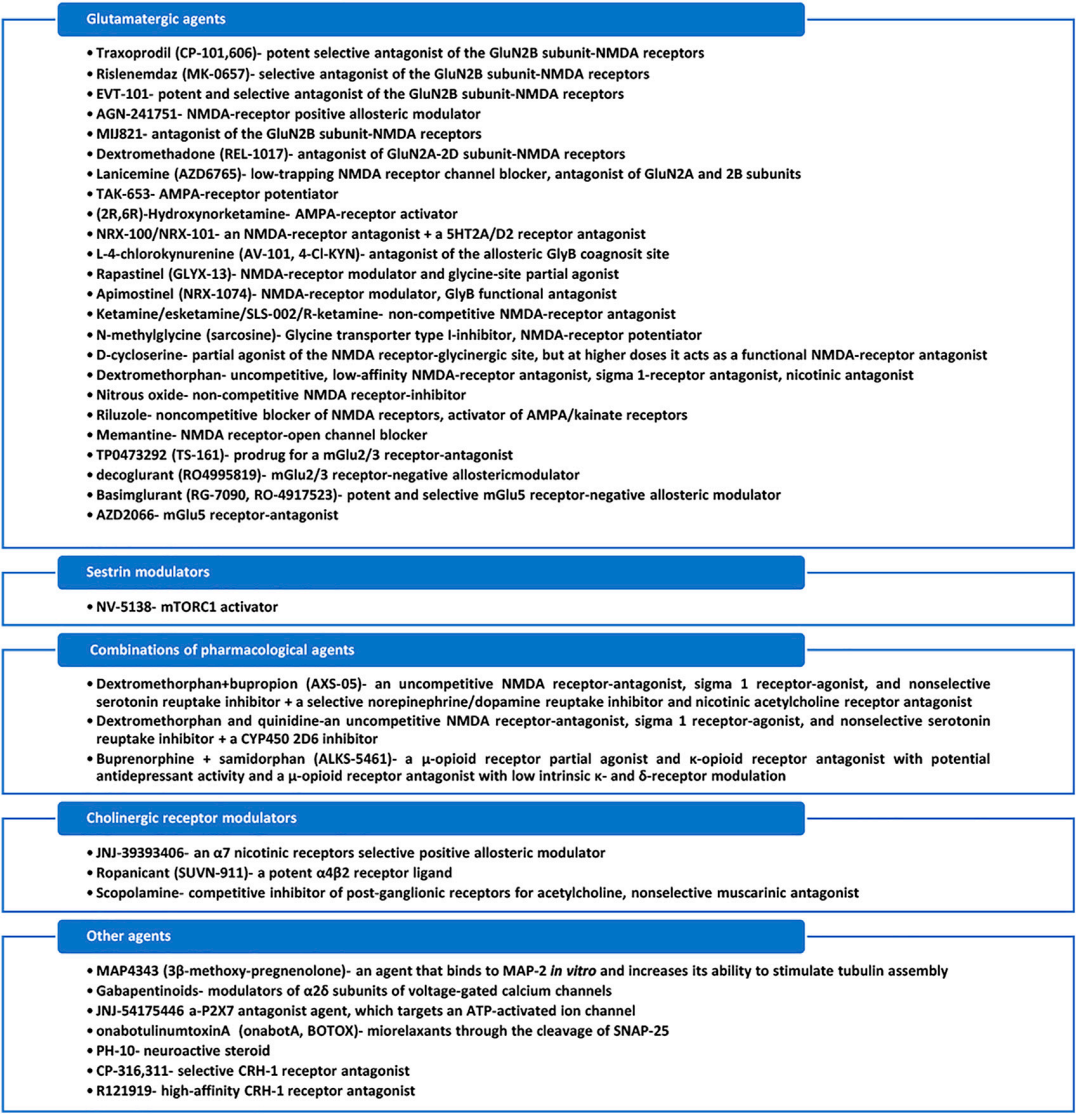
Mechanisms of action of the identified antidepressants in the pipeline, which are presented in this review.

All agents identified through this database search are presented in Figure 3.

### Glutamatergic Agents

**Traxoprodil (CP-101,606)** is a potent, selective antagonist of the GluN2B subunit within the NMDA receptor, with the capacity to potentiate the antidepressant-like effects of certain drugs in animal models (Poleszak et al., 2016). Traxoprodil inhibits the channel activity of subunits GluN1/GluN2B and reduces the time and frequency of its opening, thus preventing an excessive influx of calcium ions into neurons and secondary damage (Poleszak et al., 2016). Traxoprodil exhibited antidepressant activity in the forced swim test in rats (an animal model to screen molecules with antidepressant effect), and co-administration of traxoprodil with imipramine, fluoxetine, or escitalopram, each in subtherapeutic doses, affected at a significant level the pseudo-depressive behavior in this model (Poleszak et al., 2016).

In patients with TRD (defined by lack of response to at least one adequate trial of a selective serotonin reuptake inhibitor, SSRI), CP-101,606 was administered in a randomized, placebo-controlled, double-blind study (Preskorn et al., 2008). During the first phase of the study, subjects received a 6-week open-label administration of paroxetine and single-blind i.v. placebo infusion, with non-responders being randomized in the second phase to a double-blind single infusion of CP-101,106 or placebo plus treatment with paroxetine for up to an additional 4 weeks (Preskorn et al., 2008). The main outcome (Montgomery Asberg Depression Rating Scale, MADRS score on day 5 during the second phase) differentiated the active drug from the placebo (Preskorn et al., 2008). The response rate on Hamilton Depression Rating Scale (HAMD) was 60% *versus* 20% for traxoprodil *versus* placebo, and 78% of these active drug responders maintained their response for at least 1 week after the infusion (Preskorn et al., 2008). The antidepressant response was possible without producing significant dissociative reactions, with overall good tolerability (Preskorn et al., 2008).

A randomized, placebo-controlled, crossover pilot trial evaluated the efficacy and tolerability of the orally administered, selective GluN2B antagonist **rislenemdaz** (**MK-0657)** in patients with TRD (*N* = 5 participants) (Ibrahim et al., 2012). After 1 week drug-free period, subjects were randomized to receive either MK-0657 monotherapy (4–8 mg/day) or placebo for 12 days (Ibrahim et al., 2012). Significant antidepressant effects were reported as early as day 5 in patients receiving active drug *versus* placebo, as reflected by the evolution of the HAMD and Beck Depression Inventory (BDI) scores, but no improvement was observed on the MADRS, the primary efficacy measure (Ibrahim et al., 2012). The tolerability was good, without dissociative adverse events in patients receiving MK-0657 (Ibrahim et al., 2012).

**EVT-101** is another orally administered, potent, and selective glutamate GluN2B antagonist (Strobel et al., 2016). A phase II, randomized, double-blind, parallel-group, 4-week study was designed to evaluate the efficacy of EVT-101 in patients with TRD (after the confirmation of treatment resistance in a prospective treatment period with citalopram) but was prematurely terminated because a clinical hold was issued by FDA (NLM, NCT01128452).

**AGN-241751** is an orally active, NMDA-receptor positive allosteric modulator, currently tested as an antidepressant in clinical trials, although its precise mechanism of action and specific NMDA subunit for which it is ligand is still unknown (Pothula et al., 2021). AGN-241751 reverses behavioral deficits induced by chronic unpredictable stress in mice and possesses antidepressant-like properties in animal models (Pothula et al., 2021). Explored mechanisms of action, based on animal models, are represented by the enhancement of the NMDA-receptor activity in excitatory and parvalbumin-inhibitory neurons in the medial prefrontal cortex, activation of the Akt/mTOR signaling, and increased level of the synaptic proteins responsible for synaptic plasticity in the prefrontal cortex (Pothula et al., 2021). Also, according to the same study on mice, GluN2B subunits from the excitatory neurons in the prefrontal cortex are the initial cellular trigger underlying antidepressant effects of AGN-241751 (Pothula et al., 2021).

A two-part, double-blind, placebo-controlled, single and multiple-dose (part A) or twice-daily dose (part B), phase I/II trial conducted with adult participants (*N* = 223) diagnosed with MDD was completed in 2019 (NLM, NCT03726658). Both parts of the trial used an efficacy measure, the MADRS score, and the primary outcome was the change in this score on day 1 and day 7 after the administration of AGN-241751 (NLM, NCT03726658). No results have yet been posted as of February 2022. Another randomized, double-blind, placebo-controlled, fixed-dose, phase II trial included 251 adult participants diagnosed with MDD and evaluated the efficacy at day 1 after the initial dose of AGN-241751, defined by MADRS score change (NLM, NCT03586427). No results have been published from this trial, either.

**MIJ821** is a glutamate GluN2B antagonist investigated in a proof-of-concept, randomized, subject and investigator-blinded, parallel-group, placebo-controlled study on patients with TRD (*N* = 70 participants) (Ghaemi et al., 2021). Low dose and high dose infusions of MIJ821 (0.16 mg/kg weekly or bi-weekly *versus* 0.32 mg/kg weekly or bi-weekly) were compared to placebo (weekly) and ketamine infusion (0.5 mg/kg weekly) at 24 h, 48 h, and 6 weeks, the primary outcome measure being the change in the MADRS scores (Ghaemi et al., 2021). The adjusted mean differences *versus* placebo were significant for all MIJ821 dosing regimens and ketamine at 24 and 48 h (Ghaemi et al., 2021). At 6 weeks, none of the active interventions retained their statistical significance by comparison to placebo (Ghaemi et al., 2021).

Another double-blind, randomized, placebo-controlled, dose-ranging, phase II trial is ongoing, its objective being the investigation of efficacy and safety of intravenous MIJ821 infusion in addition to comprehensive standard of care (SOC) in patients with MDD and suicidal ideation with intent (NLM, NCT04722666). This study consists of three periods: a screening phase (up to 48 h), a double-blind core period (6 weeks), and an extension period (up to 52 weeks). It will enroll an estimate of 195 patients (NLM, NCT04722666).

**Dextromethadone (d-methadone, esmethadone, REL-1017)** has low micromolar affinity at GluN2 subunits (2A-2D) of the NMDA receptors, with a slightly superior affinity for GluN2B subunit (Callahan et al., 2004; Fogaça et al., 2019). Dextromethadone also has a very low affinity for the μ and δ-opioid receptors and does not produce opioid-like effects in humans at doses predicted to induce antidepressant activity (Fogaça et al., 2019). In a multicenter, randomized, double-blind, placebo-controlled, phase IIa trial, two dosages of REL-1017 (25 or 50 mg orally daily) were compared to placebo (*N* = 21, 19, and 22 participants, respectively) to assess the efficacy and tolerability of this product in patients with MDD who did not improve after 1–3 standard antidepressant treatments (Fava et al., 2022). Patients experienced mild or moderate adverse events during the 7 days of the trial, with no evidence of dissociative or psychotomimetic effects, opioid effects, or withdrawal signs and symptoms (Fava et al., 2022). MADRS scores improved on day 4 in both REL-1017 dosage groups, and this change persisted through the follow-up visit (day 14) (Fava et al., 2022).

Two phase III, multicenter, double-blind, placebo-controlled outpatient trials are ongoing, and they have as objective the assessment of the efficacy and safety of REL-1017 as an adjunctive treatment of MDD (RELIANCE-I, II) (NLM, NCT04688164). The estimated enrollment in these trials is estimated to be 400 participants, who will be monitored for 28 days, with changes in MADRS total score as the main outcome (NLM, NCT04855747). REL-1017 will also be evaluated as monotherapy in MDD patients in a randomized, placebo-controlled, phase III trial (RELIANCE-III) with a duration of 28 days (NLM, NCT05081167). However, another phase III trial is dedicated to the open-label evaluation of the long-term safety of REL-1017 as adjunctive treatment of MDD and is expected to recruit 600 participants for a monitoring period of 52 weeks (NLM, NCT04855760).

**AZD6765 (lanicemine)** is a low-trapping NMDA-receptor channel blocker, with an affinity for GluN2A and GluN2B complexes, with antidepressant efficacy demonstrated in three out of four clinical studies (Agbo et al., 2017; Sengupta et al., 2019). Lanicemine has a fast off-rate and is a low-trapping NMDA-receptor antagonist, unlike ketamine and MK-801 properties that lead to a favorable safety profile (Sengupta et al., 2019). This drug also acts over opiate, sigma, and muscarinic receptors (Sengupta et al., 2019). The results of two phase I studies in healthy subjects and two phase II trials in MDD patients were integrated into a pharmacokinetic analysis, and the model developed adequately described lanicemine properties in both clinical and non-clinical samples (Sanacora et al., 2014; Agbo et al., 2017). In both phase II trials, 100 mg lanicemine was efficient in decreasing the MADRS total score, and most of the secondary outcome measurements were up to 3 weeks (Sanacora et al., 2014).

In a randomized, multicenter, parallel-arm, double-blind, placebo-controlled, phase IIb trial, 302 adult patients with MDD and inadequate treatment response received 15 double-blind i.v. infusions of adjunctive lanicemine 50 mg, lanicemine 100 mg, or saline over a 12-week course, in addition to ongoing antidepressants (Sanacora et al., 2017). Lanicemine was generally well-tolerated, but neither dose was superior to placebo in decreasing the severity of the depressive symptoms (Sanacora et al., 2017).

In another double-blind, randomized, crossover, placebo-controlled trial 22 subjects diagnosed with TRD were enrolled, and they received a single infusion of AZD6765 (150 mg) or placebo on two test days, 1 week apart (Zarate et al., 2013). The MADRS score significantly improved, within 80 min, in subjects receiving AZD6765 compared to placebo, but this improvement remained significant only for 110 min (Zarate et al., 2013). The HAMD scores reflected a difference between groups at 80 and 110 min and also on day 2 (Zarate et al., 2013). The response rate was 32% in the AZD6765-treated group *versus* 15% in placebo-treated patients (Zarate et al., 2013). No difference between groups was reported in the rate of psychotomimetic and dissociative adverse effects (Zarate et al., 2013).

The contradictory results regarding the efficacy of lanicemine in phase II trials raise important questions about the drug dosage, the relevance of the placebo effect, and the potential factors that may influence treatment response in MDD patients.

TAK-653 is an AMPA receptor potentiator with virtual no agonistic activity in animal models (Hara et al., 2021). Both acute and sub-chronic administration of TAK-653 in rats produced significant antidepressant-like effects on the reduction of the submissive behavior model but did not induce a hyper locomotor response, which is a behavioral index associated with psychotomimetic side effects in humans (Hara et al., 2021).

A phase I, randomized, crossover, double-blind, placebo-controlled study enrolled 24 healthy volunteers to evaluate the central nervous system pharmacodynamic activity of TAK-653 in healthy volunteers using transcranial magnetic stimulation (TMS) (O’Donnell et al., 2021). Doses of 0.5 and 6 mg of TAK-653 or placebo were administered, and single-pulse or paired-pulse motor cortex TMS (spTMS and ppTMS) coupled with electromyography as evidence of cortical excitability change under treatment were monitored (O’Donnell et al., 2021). TAK-653 increased the amplitude of motor-evoked potentials in study participants but did not affect resting motor threshold or paired-pulse responses (O’Donnell et al., 2021). Another phase I, randomized study recruited 88 healthy subjects in order to evaluate the safety, tolerability, and pharmacokinetics of escalating single and multiple doses of TAK-653 (NLM, NCT02561156). The overall tolerability of the investigational product was good, with no severe adverse events being reported (NLM, NCT02561156).

A phase II clinical trial assessing the efficacy and safety of TAK-653 in TRD was withdrawn by the sponsor (NLM, NCT03312894).

(2R,6R)-Hydroxynorketamine is a metabolite of ketamine/esketamine, which does not bind to the NMDA receptors and does not cause dissociative effects or abuse potential in mice (Zanos et al., 2016). The antidepressant actions of hydroxynorketamine involve early and sustained AMPA-receptor activation, according to a preclinical model of depression (Zanos et al., 2016). A double-blind, placebo-controlled, phase I, single ascending dose and multiple ascending dose study focusing on the safety, pharmacokinetics, and pharmacodynamics of (2R,6R)-hydroxynorketamine in healthy volunteers is ongoing, with a total of 48 subjects planned to be enrolled (NLM, NCT04711005).

**NRX-100/NRX-101** consists of an initial single dose of ketamine (NRX-100) administered intravenously for clinical stabilization, followed by oral D-cycloserine plus lurasidone (NRX-101), and this sequential treatment regimen has as its main indication the control of suicidal ideation/behavior in bipolar depression (Hecking et al., 2021). Ketamine is an NMDA-receptor antagonist, and lurasidone is an atypical antipsychotic with 5HT2A/D2 receptor antagonist properties (Hecking et al., 2021). D-Cycloserine component of the NRX-101 is included in this combination because of its effects on inhibiting NMDA receptors and raising levels of glutamate/glutamine (Glx) in the anterior cingulate cortex (NLM, NCT03396068). Increased Glx has been reported to correlate with clinical improvement following electroconvulsive therapy and following i.v. the administration of ketamine, according to magnetic resonance spectroscopy studies (NLM, NCT03396068).

The efficacy of the sequential administration of NRX-101 has been explored in a randomized, active-comparator, phase II trial, with the main outcome being the BDM (Bipolar Inventory of Symptoms Scale-derived MADRS) score change from baseline to day 42 (NLM, NCT02974010). This trial had four arms: ketamine followed by oral NRX-101, ketamine followed by oral lurasidone, saline solution followed by oral NRX-101, and saline solution followed by oral lurasidone (NLM, NCT02974010). Many 22 adult subjects diagnosed with bipolar depression and suicidal ideation or behavior were randomized in this trial (NLM, NCT02974010). The results (yet unpublished in a peer-reviewed journal) support the superior efficacy of ketamine followed by NRX-101 *versus* ketamine followed by lurasidone, as reflected by the Bipolar Inventory of Symptoms Scale-derived MADRS (BDM) scores at day 42 (NLM, NCT02974010). No significant difference in the rate of adverse events was observed between NRX-101 and lurasidone-treated groups (NLM, NCT02974010).

NRX-101 is currently undergoing a randomized, active comparator (lurasidone), phase II trial on patients diagnosed with bipolar depression and suicidal ideation, following initial stabilization with ketamine (NLM, NCT02974010). The main outcome of this trial is the improvement of depressive symptoms as measured by MADRS total score, and the expected enrollment is 72 participants (NLM, NCT02974010). Another randomized, active comparator (lurasidone), phase II/III trial focused on the efficacy of NRX-101 in patients diagnosed with moderate bipolar depression and suicidal ideation is expected to begin recruitment, and its primary outcome will be the improvement of depressive symptoms severity measured by MADRS during 6 months (NLM, NCT03395392). A randomized, phase II/III, Glx biomarker validation study is planned to recruit 24 participants diagnosed with bipolar depression who will receive either NRX-101 *versus* placebo or NRX-101 *versus* lurasidone (NLM, NCT03402152). In this trial, the main outcome will be the mean change in the Glx area under the curve (AUC) measured after the administration of the investigational product *versus* the active comparator (NLM, NCT03402152).

The efficacy of NRX-100 (0.5 mg/kg over 40 min) is investigated in an ongoing, randomized, placebo-controlled, phase III trial, in which the primary outcome is the Columbia Suicidality Severity Scale (C-SSRS) score (NLM, NCT03396601). The main objective of this trial is to determine if NRX-100 is superior to placebo infusion in the rapid stabilization of patients with severe bipolar depression and active suicidal ideation and behavior, determined after 24 h by the percentage of participants who achieve response (C-SSRS score ≤3) (NLM, NCT03396601). Subjects who respond to NRX-100 will be offered enrollment in a 6-week follow-up study of NRX-101 *versus* SOC to validate the maintenance effect of ketamine (NLM, NCT03396601).

**L-4-Chlorokynurenine (AV-101, 4-Cl-KYN)** is an antagonist of the allosteric glycine B (GlyB) coagonist site, and this mechanism of glutamatergic modulation is considered better tolerated and safer than NMDA-receptor antagonism (Wallace et al., 2017). AV-101 is the prodrug of 7-chlorokynurenic acid, one of the most potent GlyB antagonists currently known, which possesses ketamine-like antidepressant properties in animal models of depression and efficacy in animal models of neuropathic pain (Zanos et al., 2015; Wallace et al., 2017). When the behavioral responses in animal models, measured on the 24 h forced swim test, learned helplessness test, and novelty-suppressed feeding test, were evaluated, AV-101 induced rapid, dose-dependent, and persistent antidepressant-like effects following a single dose (Zanos et al., 2015). The antidepressant effects of AV-101 were prevented by pretreatment with glycine or alpha-amino-3-hydroxy-5-methyl-4-isoxazole propionic acid (AMPA) receptor antagonists (Zanos et al., 2015). AV-101 was not associated with the rewarding or psychotomimetic effects of ketamine, and it did not lead to locomotor sensitization or stereotypic behaviors (Zanos et al., 2015).

In a randomized, controlled, double-blind, cross-over trial, the effects of AV-101 in patients with TRD were investigated (*N* = 19 participants) by the administration of 4-Cl-KYN oral monotherapy (1080 mg/day, 7 days, then 1440 mg/day, 7 days) or placebo (14 days) (Park et al., 2020). The administration of AV-101 was preceded by a period of 2 week drug-free regimen (Park et al., 2020). No treatment effects were detected using linear mixed models, as determined by primary (HAMD score) or secondary outcome measures (Park et al., 2020). No difference between groups for any peripheral or central biological indices or adverse effects was reported (Park et al., 2020). These negative results raise doubts related to the capacity of AV-101 to penetrate the brain and engage the NMDA receptors and the kynurenine pathway effectively (Murphy et al., 2021). To verify this aspect, another randomized, double-blind, placebo-controlled, crossover, phase I study (*N* = 10 healthy volunteers) explored the dose-related effects of AV-101 (720 and 1440 mg) on the engagement of the NMDA receptors (Murphy et al., 2021). The results showed that only the high dose (1440 mg) of AV-101 in humans succeeded in engaging brain targets in humans, suggesting the necessity of testing these doses in depression (Murphy et al., 2021).

**Rapastinel (GLYX-13)** is an NMDA-receptor modulator with glycine-site partial agonist properties, which possesses cognitive enhancement properties and rapid and long-lasting antidepressant activity in both animal models and humans (Burgdorf et al., 2015a). In clinical trials, rapastinel produced marked antidepressant effects that last for at least 1 week after a single dose (Moskal et al., 2014; Burgdorf et al., 2015b). Animal models of depression support the existence of a hippocampal long-term potentiation effect of rapastinel that persisted up to 2 weeks after a single dose (2 mg/kg i.v.), supposedly *via* triggering NMDA-receptor-dependent processes and increasing the mature spine density in the hippocampus and medial prefrontal cortex in rats (Burgdorf et al., 2015b).

Of the three trials identified in the clinicaltrials.gov archive, which refers to the effects of rapastinel in MDD patients, only two have results. The first proof-of-concept trial was double-blind, placebo-controlled, randomized, phase II, single i.v. GLYX-13 (1, 5, 10, or 30 mg/kg) and enrolled 116 participants with MDD who had not benefited from at least one monoaminergic antidepressant for their current episode (Preskorn et al., 2015). GLYX-13, administered at a 5 or 10 mg/kg i.v. dose reduced depressive symptoms (measured by HAMD-17) on days 1–7 (Preskorn et al., 2015). The antidepressant effect had its onset within 2 h and persisted for 7 days on average (Preskorn et al., 2015). No psychotomimetic or other significant adverse events were reported (Preskorn et al., 2015).

The second trial, with undisclosed results, included 369 participants with MDD and inadequate/partial response to antidepressants, and it had a double-blind, placebo-controlled, randomized withdrawal design (NLM, NCT01684163). A phase II, open-label extension trial investigated the safety of long-term repeat exposure to GLYX-13 in subjects who participated in the previously mentioned trial (NLM, NCT02192099; NLM, NCT01684163). In the extension, rapastinel (250/450 mg i.v.) was administered to 61 participants with completed eight or more weeks of treatment in the previous study and were willing to continue treatment (NLM, NCT02192099). Patients who were originally assigned to 5 mg/kg received 225 mg rapastinel, and those assigned to 10 mg/kg in the first trial received 450 mg active drug (NLM, NCT02192099). Unpublished results posted on the clinicaltrials.gov site show a high rate of severe adverse events (SAE) (23%) and adverse events (98%) collected during 48 months (NLM, NCT02192099). Therefore, this study was terminated by the sponsor in 32 cases, and 11 participants withdrew.

**Apimostinel (NRX-1074)** is a compound with NMDA-receptor modulating properties, more specifically, a functional antagonist at the GlyB site of the NMDA receptors (Wilkinson and Sanacora, 2019). This product was investigated in phase I trials as i.v. formulation and an orally bioavailable drug candidate (NLM, NCT02366364; NLM, NCT01856556). A phase I trial investigated the safety and tolerability of multiple oral ascending doses of NRX-1074 (375, 500, and 750 mg) in 15 healthy volunteers, but the results have not yet been released (NLM, NCT02366364). The phase I trial investigating i.v. and oral formulae also has undisclosed results (NLM, NCT01856556).

NRX-1074 led to statistically significant improvement in MDD 24 h after intravenous administration (1, 5, or 10 mg) in a randomized, double-blind, placebo-controlled phase II study (Brooks, 2015). The improvement reported after one dose of NRX-1074 infusion had an effect size (0.88), more than double the average effect size typically seen with most antidepressants after 4–6 weeks of a repeated dose, according to the company release note (Brooks, 2015). This trial recruited 140 patients with MDD, and the primary outcome measure was HAMD-17 (Brooks, 2015). It was also observed that 72% of the patients receiving the highest of the three tested doses demonstrated a clinically meaningful response at 24 h *versus* 39% in the placebo group (Brooks., 2015).

The antidepressant effects of **ketamine** are supported by randomized clinical trials, with a fast onset of action, high response rates in TRD, and efficacy against suicidality (Lacerda, 2020). While intranasal esketamine was approved by FDA in 2019 for TRD, when added to a traditional oral antidepressant, the racemic mixture of ketamine is currently investigated for MDD (Bahr et al., 2019). Ketamine/esketamine are non-competitive NMDA-glutamate receptor antagonists, with a higher affinity for these receptors in the case of S-ketamine enantiomer (Bahr et al., 2019). The mechanisms of action underlying their positive effects on MDD are unclear, but they probably involve improvement of brain plasticity *via* stimulation of BDNF (brain-derived neurotrophic factor) production and activation of the mammalian target of rapamycin (mTOR) (Sattar et al., 2018; Bahr et al., 2019). Ketamine and esketamine actions over the mTOR pathway are responsible for additional stimulation of BDNF, thus increasing brain plasticity through dendritic growth and improving synaptic transmission (Ignacio et al., 2016; Bahr et al., 2019).

A meta-analysis (*n* = 14 clinical trials) showed that a single infusion of (R,S)-ketamine (0.5 mg/g, 40 min) induces a response rate of 50%–70% in TRD (Kishimoto et al., 2016). According to a meta-analysis that evaluated the efficacy of ketamine for the treatment of MDD, the treatment effects may last up to 6 weeks after drug administration (Conley et al., 2021). Another meta-analysis that compared racemic ketamine and esketamine (*n* = 24 trials, *N* = 1877 participants) used as primary outcomes the response and remission from depression, change in depression severity, suicidality, retention in treatment, drop-out rate, and drop-outs due to adverse events, concluding that ketamine was associated with greater overall response and remission rates, as well as lower dropouts (Bahji et al., 2021).

A randomized, phase III trial evaluated the effects of i.v. ketamine (0.1, 0.25, or 0.5 mg/kg) *versus* midazolam (0.03 mg/kg) in 33 military veterans with late-life TRD (NLM, NCT02556606). The rate of response (50% reduction on MADRS total score) at day 7 was 72.7% for 0.5 mg/kg ketamine i.v. *versus* 46.2% for midazolam (active placebo) and 87.5% *versus* 66.7% at day 28 (NLM, NCT02556606).

Another interesting study evaluated the efficacy of low-dose ketamine administered during cesarean delivery as a method to decrease the incidence of postpartum depression in parturients with prenatal depression (NLM, NCT03336541). This phase IV trial was completed, but its results are not available.

SLS-002 is the racemic mixture of ketamine with intranasal administration, currently undergoing phase II clinical trials (NLM, NCT04669665). In a randomized, double-blind, crossover study, 20 TRD participants received intranasal ketamine hydrochloride (50 mg) or saline solution and were monitored for 7 days (Lapidus et al., 2014). Patients treated with ketamine had significant improvements in their depressive symptoms 24 h after drug administration, and the overall tolerability was good, with minimal adverse effects (Lapidus et al., 2014).

A phase II, randomized, initial open-label sequence and a double-blind, randomized, placebo-controlled second sequence will evaluate the efficacy, safety, and tolerability of SLS-002 in addition to SOC on symptoms of MDD and suicidality, in participants at imminent risk for suicide as determined by change in MADRS total score at 24 h after the first dose (NLM, NCT04669665). In the first part of the study, 17 patients were enrolled, and SLS-002 demonstrated a rapid onset of action from the first dose through the last visit, with the mean MADRS scores meeting the remission criteria on day 6 (PRNewswire, 2021a).

**R-Ketamine (PCN-101),** or arketamine, has been associated with a longer-lasting and more potent antidepressant effect than ketamine and esketamine in animal studies (Zhang et al., 2014). Because it proved to have weaker hypnotic and analgesic actions than the racemate and esketamine in humans, arketamine did not become commercially available for anesthesiology use (Leal et al., 2021). Unlike S-ketamine, arketamine can elicit a sustained antidepressant effect in mice, which appears to be mediated by increased BDNF-TrkB signaling and synaptogenesis in the prefrontal cortex, dentate gyrus, and CA3 hippocampal region (Yang et al., 2015). Arketamine was not associated with abuse or psychotomimetic activity (Yang et al., 2015).

In an open-label pilot trial, seven subjects with TRD received a single intravenous infusion of arketamine (0.5 mg/kg), and the MADRS score at 24 h after administration was defined as the primary outcome (Leal et al., 2021). The mean MADRS score changed significantly, with 20.3 points in 24 h, and no clear dissociative symptoms were reported (Leal et al., 2021).

A phase I, two-stage, single-center, randomized, placebo-controlled, double-blind study evaluated first the safety, tolerability, and pharmacokinetics of single PCN-101 ascending doses in 58 healthy adult volunteers, administered *via* intravenous infusion (PRNewswire, 2021b). PCN-101 was safe and well-tolerated at all doses up to 150 mg, and no SAE were reported, according to the manufacturer’s press release (PRNewswire, 2021b). In the second stage of the study, the relative safety and tolerability of PCN-101 were compared to that of S-ketamine, and the results showed that substantially higher doses of PCN-101 are required to obtain similar perceptual changes with S-ketamine (PRNewswire, 2021b).

**N-Methylglycine (sarcosine)** inhibits glycine transporter-I and thus potentiates the NMDA function, improving depression-like behavior in rodent models and depression in humans (Chen et al., 2017). A single dose of sarcosine produced an antidepressant-like effect with rapid concomitant increases in the mTOR signaling pathway activation and enhancement of the AMPA receptor membrane insertion in rats (Chen et al., 2017). Long-term administration of sarcosine had favorable effects in rats exposed to chronic unpredictable stress but not in stress-naive rats (Chen et al., 2017).

In a complex study, which explored the efficacy of sarcosine in animal models and depressed patients, the results were favorable: 1) sarcosine decreased immobility in the forced swim test and tail suspension test, reduced the latency to feed in the novelty-suppressed feeding test, and reversed behavioral deficits caused by chronic unpredictable stress test in an animal model of depression; 2) in MDD patients (*N* = 40), sarcosine (500–1500 mg/day sarcosine) improved significantly HAMD, Clinical Global Impression (CGI), and GAF scores more than citalopram (20–60 mg/day) treatment, and it was associated with a higher probability of symptom remission, quicker response, and less risk for drop out (Huang et al., 2013; NLM, NCT04975100).

A phase IV clinical trial designed to evaluate the efficacy of sarcosine as an add-on to currently administered antidepressants in patients with MDD is ongoing and is estimated to recruit 60 adult participants who will be randomized on sarcosine + SSRI or placebo + SSRI (NLM, NCT04975100). The primary outcome measure is the change in depressive symptoms severity from baseline, assessed with MADRS, during 8 weeks (NLM, NCT04975100).

**D-Cycloserine** is an antibiotic that also possesses partial agonistic properties at the NMDA-receptor-associated modulatory glycine site, and at dosages ≥100 mg/day, it acts as a functional NMDA-receptor antagonist with antidepressant effects (Heresco-Levy et al., 2006). In a double-blind, placebo-controlled 6-week crossover trial, 22 TRD patients received 250 mg/day of D-cycloserine added to their ongoing antidepressant (Heresco-Levy et al., 2006). D-Cycloserine induced symptoms reduction and was well tolerated, but the efficacy did not reach statistically significant levels in patients with D-cycloserine *versus* placebo adjuvant treatment (Heresco-Levy et al., 2006). In another double-blind, placebo-controlled, 6-week, parallel-group trial, 26 TRD patients received a gradually titrated high dose (1000 mg/day) of D-cycloserine added to their current antidepressant (Heresco-Levy et al., 2013). D-Cycloserine was well tolerated, had no psychotomimetic effects, and improved significantly depressive symptoms *versus* placebo, as measured by HAMD and BDI scores (Heresco-Levy et al., 2013). Also, pretreatment glycine serum was considered a relevant variable that interacted with the treatment outcome (Heresco-Levy et al., 2013). This second trial suggested that the antagonistic properties of D-cycloserine begin at a higher dose than expected in the first trial, probably above the level of 500 mg/day.

In another trial, 32 patients with TRD (17 with MDD and 15 with bipolar depression) who responded to ketamine infusion with an average 9.47 ± 4.11 HAMD score at baseline were randomly divided into 6-week D-cycloserine treatment *versus* placebo (Chen et al., 2019). During the 6-week treatment, the total HAMD scores did not differ between the two groups, but a potential effect of D-cycloserine over suicide ideation/behavior was identified by mixed model analysis throughout the follow-up period (Chen et al., 2019).

The administration of D-cycloserine as a pre-treatment before computer-based cognitive-behavioral therapy (CBT) sessions for depression to assess the impact of this approach on therapeutic learning has been explored in a randomized, phase II trial of 36 participants (NLM, NCT02376257). D-Cycloserine (250 mg/day) was compared in this trial with modafinil (100 mg/day) and placebo, and the primary outcome measures were the recall of CBT content, the delayed recall of emotional story items, and the delayed recall of logical memory after 2 and 3 weeks (NLM, NCT02376257). The results of this trial have not yet been published in a peer-review journal.

**Dextromethorphan** has uncompetitive, low-affinity NMDA-receptor antagonist properties and σ-1 receptor-agonist and nicotinic antagonist effects (Nguyen et al., 2016). Dextromethorphan inhibits the serotonin transporter and the norepinephrine transporter to a lesser extent (Nguyen et al., 2016). It also inhibits voltage-gated calcium channels (Nguyen et al., 2016). According to a review of the clinical and preclinical studies referring to the efficacy and tolerability of dextromethorphan, this agent is well tolerated and exerts clinically significant antidepressant effects, especially in adults with bipolar depression (Majeed et al., 2021). In a randomized, double-blind, 12-week clinical trial, 309 patients with bipolar disorder received either valproic acid and low-dose (30 or 60 mg/day) dextromethorphan or valproic acid plus placebo (Chen et al., 2014). Before treatment, patients with bipolar disorder had significantly higher plasma cytokine and lower plasma BDNF levels than healthy controls, and after treatment, HAMD and Young Mania Rating Scale (YMRS) scores in each treatment group showed significant improvement (Chen et al., 2014). Plasma cytokine levels declined in all groups, and changes in BDNF levels were significantly greater in the valproic acid + dextromethorphan 60 mg/day group than in the valproic acid + placebo group (Chen et al., 2014).

**Nitrous oxide** has a largely unknown mechanism of action, but it is considered a non-competitive inhibitor of NMDA-glutamate receptors (Kalmoe et al., 2020). Its main clinical use is inhalational general anesthesia and analgesia for short procedures, but it is also used recreationally by adolescents and young adults (Kalmoe et al., 2020). The euphoria-inducing effects of nitrous oxide have been hypothesized to have clinical benefits in patients with MDD (Kalmoe et al., 2020). In a proof-of-concept, placebo-controlled crossover trial, 20 patients with TRD were randomized to 1 h inhalation of 50% nitrous oxide/50% oxygen or 50% nitrogen/50% oxygen (the last one being equivalent to placebo) (Nagele et al., 2015). Depressive symptoms improved significantly at 2 and 24 h after nitrous oxide administration *versus* placebo (according to HAMD-21 scores) (Nagele et al., 2015). Treatment response was observed in four patients (20%), and three patients had a full remission after nitrous oxide *versus* one patient (5%) and none after placebo (Nagele et al., 2015). No SAE occurred, and all adverse events were brief and of mild-to-moderate severity (Nagele et al., 2015). Another phase II, randomized, double-blind trial that evaluated the efficacy of inhaled nitrous oxide for TRD investigated the impact of nitrous oxide 25% or 50% *versus* placebo over HAMD-21 scores at 2 and 24 h after inhalation in 34 patients, but results have not been disclosed (NLM, NCT03283670).

**Riluzole** is a neuroprotective agent which inhibits the voltage-dependent sodium channels on glutamatergic nerve terminals and activates AMPA/kainate receptors, but it may induce a noncompetitive blockade of NMDA receptors (Doble, 1996; Zarate et al., 2004).

In an open-label trial, 19 patients diagnosed with treatment-resistant depression received riluzole 168.8 mg/day (mean dose) for 6 weeks (Zarate et al., 2004). Significant improvement in MADRS scores occurred in weeks 3–6, in trial completers, and CGI-S and HAMA also improved significantly during weeks 3–6 (Zarate et al., 2004). The response rate for completers at week 6 was 46%, and the remission rate was 31% (Zarate et al., 2004). The most common adverse events during the trial were headache (58%), gastrointestinal distress (43%), tension, or inner unrest (26%) (Zarate et al., 2004).

In an open-label trial, 100–200 mg riluzole was administered for 6 weeks to 14 patients with bipolar depression, and it led to a significant reduction of HAMD scores, while the glutamine/glutamate (Gln/Glu) ratios increased significantly by day 2 of the treatment (Brennan et al., 2010). N-Acetyl aspartate (NAA) levels increased in NAA from baseline to week 6 (Brennan et al., 2010). Therefore, riluzole seems to rapidly increase the Gln/Glu ratios, suggesting increased glutamate-glutamine cycling, which may lead to enhanced neuronal plasticity and reduced depressive symptoms (Brennan et al., 2010).

Riluzole was added to ongoing medication for 6 weeks, followed by an optional 6-week continuation phase in 10 patients diagnosed with treatment-resistant depression (Sanacora et al., 2007). HAMD and HAMA scores declined significantly following the initiation of riluzole augmentation treatment, and the effect of riluzole became significant at the end of the first week of the trial and persisted for the 12-week duration of monitoring (Sanacora et al., 2007).

A phase II, randomized, double-blind, placebo-controlled, adjunctive trial on treatment-resistant MDD enrolled 104 participants who received 1) 100 mg riluzole added to ongoing SSRI/SNRI for 8 weeks, 2) riluzole/placebo added to SSRI/SNRI for 4 weeks and placebo added to the same agents for another 4 weeks, or 3) placebo added to SSRI/SNRI for 8 weeks (NLM, NCT01204918). The main outcome measures were the change in MADRS scores after 4 and 8 weeks (NLM, NCT01204918). The final results of this trial were not published in a journal, but the rough data available on the clinicaltrials.gov site did not support a large difference between groups, while the response rate at week 8 (secondary outcome) was higher for placebo than for any of the active groups (NLM, NCT01204918).

Another randomized, double-blind, phase II trial evaluated the efficacy of riluzole (50 mg b.i.d) *versus* placebo as an add-on to sertraline (100 mg/day) in 21 outpatients diagnosed with MDD during 8 weeks, and the primary outcome measures were the mean change in HAMD scores from baseline to endpoint and the number of patients with antidepressant response or remission at week 8 (NLM, NCT01703039). This study was prematurely terminated due to administrative reasons.

Another 6-week, single-arm, single-blind phase II study enrolled 31 patients with MDD without psychotic features and evaluated the efficacy of riluzole (NLM, NCT00026052). The study was completed, but no results were posted or published.

A randomized, placebo-controlled, double-blind, continuation-phase IV study evaluated the safety and effectiveness of ketamine and riluzole in patients with treatment-resistant MDD (Mathew et al., 2010). A total of 26 medication-free patients received open-label i.v. ketamine (0.5 mg/kg over 40 min), and before infusion, they were randomized to lamotrigine (300 mg) or placebo (Mathew et al., 2010). The response rate was 65% (17 patients), according to the MADRS scores at 24 h following ketamine, while lamotrigine failed to attenuate the mild, transient side effects associated with ketamine and did not enhance its antidepressant effects (Mathew et al., 2010). After 72 h of infusion, the response was obtained by 14 patients (54%), and they were randomized to continue with riluzole (100–200 mg/day) or placebo (Mathew et al., 2010). An interim analysis did not find any significant differences between riluzole and placebo regarding the main outcome (time-to-relapse), with 80% of patients relapsing on riluzole *versus* 50% on placebo (Mathew et al., 2010). Therefore, the trial was discontinued for futility.

A randomized, placebo-controlled, double-blind, phase II trial evaluated the efficacy and safety of riluzole (50–200 mg/day) in 94 participants diagnosed with bipolar depression for 8 weeks, and the main outcome measure was the mean change in MADRS score (NLM, NCT00376220). The results were posted on clinicaltrials.gov and did not support the superior efficacy of riluzole *versus* placebo (NLM, NCT00376220).

Another 8-week, open-label study of riluzole (50–200 mg/day) in combination with lithium recruited 14 acutely depressed bipolar patients (MADRS score ≥20) who first followed 4 weeks of lithium treatment (Zarate et al., 2013). The linear mixed model for total MADRS score showed a significant treatment effect at week 8, without cases of switch into hypomania or mania (Zarate et al., 2013).

An 8-week, double-blind, placebo-controlled, phase II trial evaluated the efficacy and safety of riluzole (50–200 mg/day) in 19 participants diagnosed with bipolar depression, but the study was terminated due to the superior efficacy of placebo in an interim analysis (NLM, NCT00054704).

**Memantine** is classified as an NMDA-receptor-open channel blocker because it can enter these channels and block current flow only after they are opened (Johnson and Kotermanski, 2006). A double-blind, placebo-controlled trial enrolled 32 patients diagnosed with MDD, randomized on memantine (5–20 mg/day) or placebo for 8 weeks (Zarate et al., 2006). The results of this trial did not support the efficacy of memantine based on the linear mixed models for total MADRS scores (Zarate et al., 2006). Another randomized, double-blind, placebo-controlled trial evaluated the efficacy of memantine (5–20 mg/day) as an add-on to antidepressant treatment in 31 participants with partial or non-responsive MDD for 8 weeks (Smith et al., 2013). No significant change in MADRS scores was detected in patients who received memantine *versus* those on placebo, either over the entire study or at study completion (Smith et al., 2013). A minimal-to-small effect size was observed, favoring placebo (d = 0.19) (Smith et al., 2013). No statistical differences were observed between groups on secondary efficacy outcomes or safety outcomes (Smith et al., 2013).

A phase IV, randomized, placebo-controlled trial investigated the efficacy and safety of memantine (5–20 mg) augmentation administered for 8 weeks in 29 adult patients diagnosed with bipolar depression and incomplete response to lamotrigine (NLM, NCT00305578). The primary outcome was the change in HAMD-17 from baseline to week 8, and the posted results on clinicaltrials.gov show a decrease of 9 *versus* 7 points in patients treated with memantine *versus* placebo (NLM, NCT00305578). The most frequently reported adverse events in the memantine group were somnolence, indigestion, diarrhea, headache, and coughing (NLM, NCT00305578).

A double-blind, randomized, phase III trial evaluating the safety and effectiveness of memantine (5–20 mg/day) included three phases: during the first stage, adult outpatients with MDD without psychotic features (*N* = 112) have tapered off all psychiatric medications over 2 weeks (washout period); in the second phase, participants were randomized on memantine or placebo three times a day for 8 weeks; and participants who responded well to the treatment entered phase III, a 16-week continuation period of either memantine or placebo (NLM, NCT00040261). No results of this trial have been released.

However, another single-site, double-blind, placebo-controlled, parallel-group, phase IV trial enrolled 31 participants diagnosed with MDD and non-response or incomplete response to antidepressants were randomized on either memantine (5–20 mg/day) or placebo as an add-on for 8 weeks (NLM, NCT00344682). The main outcome was the change in MADRS scores at week 8, and the results posted on clinicaltrials.gov did not support a significant difference between groups (−7.13 *vs*. −7.25 points in memantine *vs*. placebo) (NLM, NCT00344682). The rate of serious adverse events was similar in the two groups (20 *vs*. 18.75% in memantine *vs*. placebo) (NLM, NCT00344682).

The blockade of metabotropic glutamate 2/3 (mGlu2/3) receptors is considered a potentially interesting approach in the treatment of MDD, based on several preclinical studies (Sanacora et al., 2008; Watanabe et al., 2021). **TP0473292 (TS-161)** is the prodrug of a novel mGlu2/3 receptor antagonist, investigated in trials for MDD treatment (Watanabe et al., 2021). In a first-in-human, randomized, double-blind, single ascending dose (15–400 mg) and 10-day-multiple-ascending dose (50–150 mg), phase I trial on healthy subjects (*N* = 70), the pharmacokinetic profile of TS-101 was described (Watanabe et al., 2021). The prodrug was extensively converted into its active metabolite, and plasma exposure to this metabolite increased with the dose administered (Watanabe et al., 2021). The investigational product penetrated the brain–blood barrier, and the most frequently reported adverse events were nausea, vomiting, and dizziness, with an exposure-related incidence (Watanabe et al., 2021). An ongoing, placebo-controlled, phase II study is dedicated to the evaluation of TS-161 efficacy in TRD, with the main outcome being the change from baseline to day 21 on MADRS total scores and an estimated enrollment of 25 participants (NLM, NCT04821271).

On the contrary, **decoglurant (RO4995819)**, a mGlu2/3 receptor negative allosteric modulator, failed in a phase II trial to exert any antidepressant or procognitive effects (Umbricht et al., 2020). During this 6-week, double-blind, multicenter, randomized trial, 357 participants diagnosed with MDD who did not respond to two adequate trials of an SSRI/SNRI received decoglurant 5 mg (*N* = 101), 15 mg (*N* = 102), or 30 mg (*N* = 55) daily, or placebo (*N* = 99) although their adherence was confirmed through positive drug levels (Umbricht et al., 2020). At week 6, no significant differences were observed between any active treatment arms and placebo in decreasing MADRS scores, in response, or in remission rates (Umbricht et al., 2020). No effects of decoglurant were observed on Cambridge Neuropsychological Test Automated Battery (CANTAB)—cognitive accuracy and cognitive speed composite scores (Umbricht et al., 2020). High placebo response was observed, which may impair the ability of this trial to detect an efficacy signal. Another phase II trial that was intended to evaluate the efficacy of decoglurant *versus* placebo as adjunctive therapy in patients with MDD and inadequate response to their ongoing antidepressant was withdrawn by the sponsor (NLM, NCT01733654).

Metabotropic glutamate type 5 (mGlu5) receptors are ubiquitously expressed throughout the brain, and their dysfunction is involved in the pathogenesis of several diseases, for example, Alzheimer’s disease, Parkinson’s disease, and MDD (NLM, NCT01145755). Despite the success of the negative allosteric mGlu5 receptor modulators in preclinical studies, no such agent has been associated with favorable results in clinical trials with MDD patients (NLM, NCT01145755). **Basimglurant (RG-7090, RO-4917523)** is a potent and selective mGlu5 receptor negative allosteric modulator with good oral availability and a long half-life, which allows for once-daily administration (Lindemann et al., 2015; Quiroz et al., 2016). It has antidepressant properties and anxiolytic-like and antinociceptive effects (Lindemann et al., 2015). In a phase IIb, multicenter, double-blind, randomized clinical trial, basimglurant MR (0.5 or 1.5 mg) was compared with placebo in 333 adult patients with MDD, as adjunctive to ongoing antidepressant medication (an SSRI or SNRI agent), for 6 weeks (Quiroz et al., 2016). No difference was observed in the primary outcome, MADRS change from baseline to the endpoint, between basimglurant MR and placebo (Quiroz et al., 2016). Secondary endpoints were modified by adjunctive basimglurant MR 1.5 mg daily, especially in patient-rated measures (Quiroz et al., 2016). The most frequently reported adverse event was dizziness, but it was of mild intensity and transient (Quiroz et al., 2016). Another phase I, single-center, randomized, multiple-ascending dose trial, evaluated the safety of basimglurant *versus* placebo (*N* = 56 participants with MDD or healthy subjects) (NLM, NCT02433093). No results of this trial were posted as of February 2022.

**AZD2066** is a mGlu5 receptor antagonist that was assessed in a phase IIa, multicenter, randomized, double-blind, double-dummy, active (duloxetine), placebo-controlled, and parallel-group study on 131 patients diagnosed with MDD, and the results (posted on clinicaltrials.gov) were negative (MADRS total score change was the primary outcome) (Arsova et al., 2020; NLM, NCT01145755).

In conclusion, glutamate modulators are a promising class of antidepressant agents, although several molecules have failed in different stages of clinical development. The recent FDA approval of intranasal esketamine as an adjunctive agent for TRD is an argument in favor of glutamatergic neurotransmission importance in the pathophysiology of mood disorders.

### Sestrin Modulators

Sestrins are small, stress-induced proteins with multiple roles; for example, they are involved in oxidative stress, DNA damage response, cell growth, metabolic homeostasis, and mTORC1 signaling (Sengupta et al., 2019). The inhibition of mTORC1 by sestrins 1 and 2 can be reversed by the influx of sufficient levels of amino acids, whereas sestrin 3 cannot be regulated by amino acids (Sengupta et al., 2019). Suppressed mTORC1 signaling has been suggested as a possible pathogenic mechanism in MDD, and NMDA receptor modulators such as ketamine are dependent upon the mTORC1 activation in brain areas responsible for mood, for example, the medial prefrontal cortex (Sengupta et al., 2019).

**NV-5138** is a novel small molecule activator of mTORC1 signaling, both *in vivo* and *in vitro*, orally available, and can transiently activate mTORC1 in several peripheral tissues and the brain (Sengupta et al., 2019). The impact of NV-5138 on synaptic function and BDNF signaling is similar to ketamine, suggesting a shared mTORC1 signaling-mediated mechanism for their antidepressant effect (Kato et al., 2019). A single dose of NV-5138 produced a rapid and long-lasting antidepressant effect and rapidly reversed anhedonia caused by chronic stress exposure in animal models of depression (Kato et al., 2019). The antidepressant action of NV-5138 required BDNF release, as the behavioral responses were blocked by infusion of BDNF-neutralizing antibodies into the medial prefrontal cortex (Kato et al., 2019).

A randomized, placebo-controlled, phase I trial explored the effects of a single ascending dosage level of NV-5138 in healthy volunteers and a single dose of NV-5138 in subjects with TRD, but no results of this trial have yet been disclosed (NLM, NCT03606395).

Another placebo-controlled, randomized, phase II trial is planned to evaluate the efficacy and tolerability of NV-5138 in adults with TRD (estimated enrollment: 40 participants), with a monitoring period of 5 weeks, and MADRS change to baseline as the primary outcome measure (NLM, NCT05066672).

In conclusion, the modulation of sestrins as a pharmacodynamic substrate for a new class of antidepressants is still in the early phases of research (Table 2) and is supported mostly by animal studies.

**TABLE 2:** Sestrin modulators with antidepressant properties in the pipeline.

Authors	Methodology	Results	Clinical trial phase, trial identifier (if available)
NLM (2018g)	NV-5138 *vs.* placebo, SAD, DBRCT, N_1_ = 48 (estimated) healthy volunteers and N_2_ = 40 treatment-resistant MDD, 9 days	The primary outcome was the incidence of treatment-related AE. The results of this trial have not yet been disclosed	Phase I, NCT03606395
NLM (2021h)	NV-5138 *vs.* placebo, DBRCT, *N* = 40 (estimated), TRD patients, 5 weeks	The primary outcome measure was the efficacy measured by MADRS total score. This trial is ongoing	Phase II, NCT05066672

AE, adverse events; DBRCT, double-blind, randomized controlled trial; MADRS, Montgomery Asberg Depression Rating Scale; MDD, major depressive disorder; SAD, single ascending dose; TRD, treatment-resistant MDD.

### Combinations of Pharmacological Agents

**The combination of dextromethorphan and bupropion (AXS-05)** is currently explored as an orally administered therapy for patients with MDD, based on the pharmacodynamic properties of an uncompetitive NMDA-glutamate antagonist, σ1 agonist, and nonselective serotonin reuptake inhibitor (dextromethorphan), and a selective norepinephrine/dopamine reuptake inhibitor with nicotinic acetylcholine receptor antagonist properties (bupropion) (Sakurai et al., 2022). Beside its antidepressant properties, bupropion is credited with the protection of dextromethorphan from rapid metabolization *via* CYP450 2D6 because of this antidepressant potent inhibition of these hepatic isoenzymes (Sakurai et al., 2022).

In a randomized, double-blind, active-controlled, multicenter phase II trial (ASCEND), 80 patients diagnosed with moderate-to-severe MDD were treated for 6 weeks with AXS-05 (45 mg dextromethorphan/105 mg bupropion twice daily) or bupropion (105 mg twice daily) (Axsome Therapeuticals, 2019). Change in MADRS score was the primary outcome, and the rate of remission and response was superior for the AXS-05 group at the end-point, with early separation from the bupropion-treated group (Axsome Therapeuticals, 2019). The pharmacological combination was safe and well-tolerated, with similar rates of adverse events in the AXS-05 and bupropion arms (Axsome Therapeuticals, 2019). In the AXS-05 group, the most frequent adverse events were nausea, dizziness, dry mouth, decreased appetite, and anxiety (Axsome Therapeuticals, 2019). No psychotomimetic effects, weight gain, or increased sexual dysfunction were reported in the AXS-05 group (Axsome Therapeuticals, 2019).

Another phase II, randomized, double-blind, placebo-controlled, relapse prevention, multicenter trial (MERIT) explored the efficacy of AXS-05 in patients with TRD (*N* = 44 participants, who presented ongoing symptoms of depression despite receiving treatment with two or more prior antidepressants during the current major depressive episode) (Axsome Therapeuticals, 2021). Patients who achieved stable remission under AXS-05 in a previous trial (MADRS scores ≤ 12 at two or more consecutive visits, separated by at least 4 weeks) were randomized to continue the same treatment or to discontinue it and switch to placebo (Axsome Therapeuticals, 2021). AXS-05 met the primary endpoint by significantly delaying the time to relapse of depressive symptoms compared to placebo, with no relapse over ≥6 months (Axsome Therapeuticals, 2021). Also, AXS-05 met the key secondary endpoint of relapse prevention, according to the relapse rates during the double-blind period (Axsome Therapeuticals, 2021).

In a phase III randomized, double-blind, placebo-controlled, multicenter trial (GEMINI), 327 adult patients diagnosed with moderate-to-severe MDD were randomized to treatment with either AXS-05 or placebo once daily for the first 3 days and twice daily thereafter for a total of 6 weeks (Axsome Therapeuticals, 2020). AXS-05 demonstrated a significant reduction in patient-reported depressive symptoms, evaluated by QIDS-SR-16 and PGI-I (Patient Global Impression of Improvement), compared to placebo at week 6 (Axsome Therapeuticals, 2020). The response on QIDS-SR-16 total score (at least 50% improvement) was significantly greater for AXS-05 starting from week 1 and at every time point thereafter, with 53.4% of patients achieving response compared to 33% of placebo patients at week 6 (Axsome Therapeuticals, 2020). On the PGI-I, AXS-05 demonstrated efficacy *versus* placebo, with 47.2% of patients achieving the level of “very much” or “much” improvement *versus* 31.3% of placebo patients at week 6 (Axsome Therapeuticals, 2020). The evolution of symptoms measured with clinician-rated scales (i.e., MADRS and CGI-I) supported the favorable results observed on self-reported scales, the difference from placebo being consistent at week 6 (Axsome Therapeuticals, 2020). The most commonly reported adverse events associated with AXS-05 were dizziness, nausea, headache, diarrhea, somnolence, and dry mouth (Axsome Therapeuticals, 2020).

Another phase III, randomized, double-blind, active-controlled, multicenter trial (STRIDE-1) evaluated the efficacy of AXS-05 in TRD (*N* = 312 adult participants who had failed two or three prior treatments) but did not show a statistically significant difference between the investigational product (45 mg dextromethorphan/105 mg bupropion, twice daily) and active control (150 mg bupropion, twice daily) after 6 weeks, according to MADRS total score (Biospace, 2020). The secondary outcomes favored, however, AXS-05 *versus* active control, with significantly higher rates of remission from depression (defined by QIDS-SR-16 ≤ 5) at week 1 and at every time point thereafter (Biospace, 2020). Also, AXS-05 improved cognitive function (monitored by the Massachusetts General Hospital Cognitive and Physical Functioning Questionnaire, CPFQ) and reduced anxiety symptoms (Hamilton Anxiety Rating Scale, HAM-A) (Biospace, 2020).

**The fixed-dose combination of dextromethorphan hydrobromide and quinidine** (DXMQ) was created based on the CYP450 2D6 enzyme inhibition induced by quinidine and the previously mentioned pharmacodynamic properties of dextromethorphan, which recommend this combination as a potential antidepressant therapy (Murrough et al., 2017; Majeed et al., 2021). DXMQ was approved by FDA in 2010 for the treatment of the pseudobulbar affect (Murrough et al., 2017). In an open-label, phase IIa clinical trial examining the efficacy and tolerability of orally administered DXMQ (45 mg/10 mg every 12 h) in 20 patients with TRD during 10 weeks, the MADRS score (primary outcome) significantly decreased from baseline to endpoint (Murrough et al., 2017). The QIDS-SR score also decreased significantly during the DXMQ treatment, and the response and remission rates in the intent-to-treat sample were 45% and 35%, respectively (Murrough et al., 2017).

A retrospective chart review included depressed patients (*N* = 77) diagnosed with treatment-resistant bipolar disorder type II or NOS, who received treatment with DXMQ 20 mg/10 mg twice daily in addition to their current treatment (Kelly and Lieberman, 2014).On day 90, the CGI-I score was 1.66, and some patients improved their clinical status within 1–2 days after the beginning of DXMQ administration (Kelly and Lieberman, 2014). A significant number of patients (*N* = 19) discontinued treatment due to adverse events, mainly nausea (Kelly and Lieberman, 2014).

**FIGURE 4:**
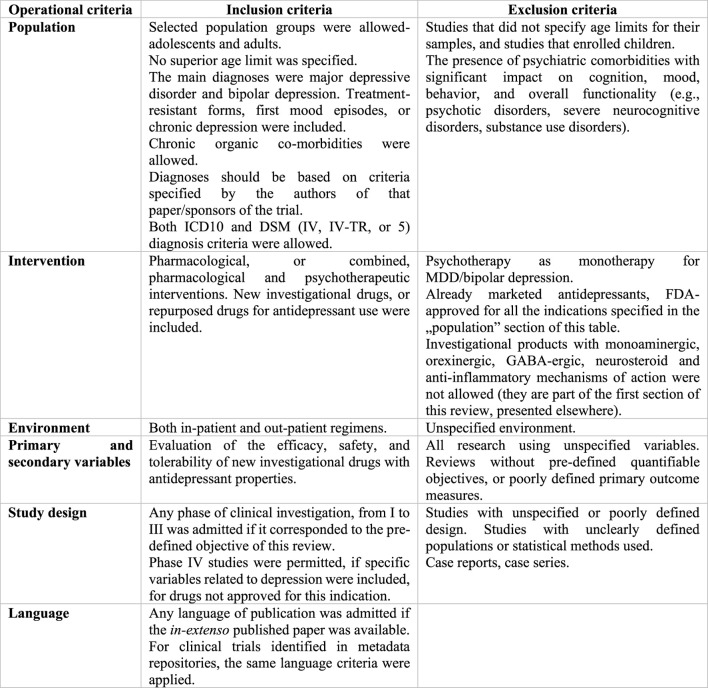
Inclusion and exclusion criteria.

**Deudextromethorphan/quinidine (AVP-786)** combines d6-dextromethorphan and quinidine sulfate in an oral formulation, with deuterium in the dextromethorphan molecule, a heavier and stable isotope of hydrogen, in order to increase this drug’s half-life (Gant, 2014). A phase II, multicenter, randomized, double-blind, placebo-controlled study assessed the efficacy, safety, and tolerability of DXMQ as adjunctive therapy in patients with MDD and inadequate response to antidepressant treatment (*N* = 206 participants) (NLM, NCT02153502). The primary outcome was the change in the MADRS score during the 10 weeks of the trial (NLM, NCT02153502). No results have been yet posted as of February 2022 (Figure 4).

The co-formulation of **buprenorphine and samidorphan (ALKS-5461)** associates a μ-opioid receptor partial agonist and κ-opioid receptor antagonist with potential antidepressant activity (buprenorphine) and a μ-opioid receptor antagonist with low intrinsic κ- and δ-receptor modulation (samidorphan), which is intended to decrease the risk of buprenorphine abuse and dependence (Thase et al., 2019).

A phase II, multicenter, randomized, double-blind, placebo-controlled, two-stage sequential parallel comparison trial enrolled adults with MDD who had an inadequate response to 1–2 courses of antidepressant treatment (Fava et al., 2016). Participants (*N* = 142) were randomized to adjunctive treatment with 2/2 mg BUP/SAM, 8/8 mg BUP/SAM, or placebo, and they were monitored using HAMD, MADRS, and the CGI-S for 4 weeks, then they followed a 1-week taper (Fava et al., 2016). Compared to the placebo group, significant improvements were reported in patients treated with 2/2 mg BUP/SAM across the three depression outcome measures, and evidence of improvement was also found in the 8/8 mg BUP/SAM group, but without achieving statistical significance (Fava et al., 2016). The overall tolerability was good, and there was no evidence of opioid withdrawal when treatment was discontinued (Fava et al., 2016).

In the FORWARD-3 trial, adult patients with MDD and inadequate response to antidepressant therapy (*N* = 399 participants in group 1 and 30 in group 2) were randomized in a double-blind manner to 2/2 mg bupropion/samidorphan (BUP/SAM) or placebo for 6 weeks (Zajecka et al., 2019). There were no differences in the MADRS-based response or remission rates between groups, and the least-square mean change in the MADRS total score at the end of treatment was not significantly different from placebo, although BUP/SAM did improve the overall depressive symptoms severity (Zajecka et al., 2019). Adverse events were mild or moderate in severity, and no evidence of abuse potential during treatment was detected (Zajecka et al., 2019).

Two global, multicenter, randomized, double-blind, placebo-controlled, sequential parallel-comparison design studies (FORWARD-4 and FORWARD-5) evaluated the safety and tolerability of 2/2 mg ALKS-5461 as an adjunctive treatment for MDD in adults who did not present an adequate response to antidepressant therapy (N_1_ = 385 participants, and N_2_ = 407 participants) (Fava et al., 2020). FORWARD-4 also evaluated a 0.5/0.5 mg dose and FORWARD-5 a 1/1 mg dose for 5 weeks during the first stage and 6 weeks during the second stage (Fava et al., 2020). FORWARD-5 achieved the primary endpoint because 2/2 mg BUP/SAM was superior to placebo, according to the MADRS total score and MADRS-6 (Bech) score change from baseline to the last visit (Fava et al., 2020). However, FORWARD-4 did not achieve the primary endpoint, although separate analyses showed significant differences between groups at other time points (Fava et al., 2020). The pooled analysis of these two trials demonstrated a greater reduction in MADRS total scores from baseline for 2/2 mg BUP/SAM *versus* placebo at multiple time points, including the last visit, and a significant average change from baseline to week 3 to the end of the study (Fava et al., 2020). The overall tolerability of BUP/SAM was good, with most adverse events being of mild or moderate severity. There was minimal evidence of abuse and no evidence of dependence or opioid withdrawal by adverse events report or objective measures (Fava et al., 2020). FORWARD-1 was a phase III, randomized, double-blind trial that evaluated the safety and tolerability of two titration regimens for ALKS-5641 as adjunctive treatment in MDD adults with inadequate response to antidepressant therapy (*N* = 66 patients) (NLM, NCT02085135). No results were published, but according to the raw data presented on the clinicaltrial.gov archive, no SAE were recorded in either group, while 67.65% of the subjects who received 1-week titration and 87.5% of those with 2-week titration had adverse events during the 8 weeks of monitoring (NLM, NCT02085135).

FORWARD-2 was an open-label, 52-week study to evaluate the long-term safety and tolerability of BUP/SAM 2/2 mg as adjunctive therapy to ongoing antidepressant treatment for MDD patients unresponsive to prior antidepressant therapy (*N* = 1486 participants) (Thase et al., 2019).

Adverse events were reported by 75.7% of the patients, but the majority were of mild or moderate intensity (Thase et al., 2019). The most common adverse events were nausea, headache, constipation, and dizziness (Thase et al., 2019). Discontinuation due to adverse events was recorded in 10.4% of the cases, and SAE were reported in 3.2% of the patients (Thase et al., 2019). Following abrupt BUP/SAM discontinuation, the incidence of opioid withdrawal symptoms was low (6.5%) (Thase et al., 2019). Improvements in MADRS scores were maintained until the last visit, suggesting durability of antidepressant effect in patients receiving continuous treatment (Thase et al., 2019).

Another randomized, placebo-controlled, double-blind, phase IIIb trial evaluated the efficacy, safety, and tolerability of adjunctive ALKS-5461 in patients with treatment-refractory MDD (*N* = 278 participants) (NLM, NCT03188185). This study had a sequential parallel comparison design: in stage 1, subjects were randomized to ALKS-5461 or placebo, and in stage 2, only placebo non-responders from stage 1 were re-randomized to active drug or placebo (NLM, NCT03188185). The results posted on clinicaltrials.gov show non-significant differences between groups according to the main outcome measure, MADRS score (*p* = 0.128) (NLM, NCT03188185). The overall tolerability was good, with no SAE recorded in either stage of this trial, while the most reported adverse events within the ALKS-5461-treated patients were nausea, constipation, vomiting, fatigue, dizziness, somnolence, headache, and sedation (NLM, NCT03188185).

In conclusion, various formulations of combined pharmacological agents have been investigated for MDD or TRD, with positive results for AXS-05 (although a negative phase III trial also exists) and DXMQ (a single-phase IIa trial) but controversial results for ALKS-5461 (phase II and III trials) (Table 3).

**TABLE 3:** Combinations of pharmacological agents with antidepressant properties in the pipeline.

Authors	Methodology	Results	Clinical trial phase, trial identifier (if available)
NLM (2019b)	AXS-05 *vs*. bupropion, DBRCT, *N* = 80 moderate-to-severe MDD patients, 6 weeks	Change in the MADRS score was the primary outcome, and the rate of remission and response was superior for the AXS-05 group at the end-point, with early separation from the bupropion-treated group. The pharmacological combination was safe and well-tolerated. The most frequent treatment-related AE were nausea, dizziness, dry mouth, decreased appetite, and anxiety	Phase II, NCT03595579
NLM (2021i)	AXS-05 *vs*. placebo, DBRCT, *N* = 44, TRD patients, 52 weeks	AXS-05 met the primary endpoint by significantly delaying the time to relapse of depressive symptoms compared to placebo, with no relapse over at least 6 months Also, the active medication met the key secondary endpoint of relapse prevention	Phase II, NCT04608396
NLM (2020)	AXS-05 *vs*. placebo, DBRCT, *N* = 327 moderate-to-severe MDD patients, 6 weeks	AXS-05 significantly decreased patient-reported depressive symptoms, evaluated by QIDS-SR-16 and PGI-I, compared to placebo at week 6. The response on QIDS-SR-16 total score was significantly greater for AXS-05 starting from week 1 and at every time point thereafter, with 53.4% of patients achieving response compared to 33% of placebo patients at week 6	Phase III, NCT04019704
Biospace (2020)	AXS-05 *vs*. bupropion, DBRCT, *N* = 312 TRD patients, 6 weeks	The change in MADRS total score was not significantly different between the two groups. The secondary outcomes favored, however, AXS-05 *vs*. active control, with significantly higher rates of remission from depression at week 1 and at every time point thereafter. AXS-05 improved cognitive function and reduced anxiety symptoms	Phase III, NCT02741791
Murrough et al. (2017)	DXMQ, open-label, *N* = 20 TRD patients, 10 weeks	MADRS score (primary outcome) significantly decreased from baseline to endpoint. The QIDS-SR score also decreased significantly during DXMQ treatment, and the response and remission rates in the intent-to-treat sample were 45% and 35%, respectively	Phase IIa, NCT01882829
Kelly and Lieberman (2014)	DXMQ + ongoing antidepressant, retrospective, *N* = 77 treatment-resistant BD type II/NOS patients	On day 90, the CGI-I score was 1.66, and some patients improved their clinical status within 1–2 days after the beginning of DXMQ administration. An important number of patients (*N* = 19) discontinued treatment due to AE, mainly nausea	Phase N/A
NLM (2014a)	AVP-786 *vs*. placebo as adjunctive to current therapy, DBRCT, *N* = 206 MDD patients with inadequate response to antidepressants, 10 weeks	The primary outcome was MADRS total score. Undisclosed results	Phase II, NCT02153502
Fava et al. (2016)	ALKS-5461 *vs*. placebo, DBRCT, *N* = 142 MDD patients with inadequate response to antidepressant therapy, 5 weeks	Significant improvements were reported in patients treated with 2/2 mg BUP/SAM in HAMD, MADRS, and CGI-S, and evidence of improvement was also found in the 8/8 mg BUP/SAM group, but without achieving statistical significance. The overall tolerability was good	Phase II, NCT01500200
Zajecka et al. (2019)	ALKS-5461 *vs*. placebo as adjunctive therapy, *N* = 399 + 30, MDD patients with inadequate response to antidepressant treatment, 6 weeks	There were no differences in MADRS-based response or remission rates between groups, and the LSM change in MADRS total score at the end of treatment was not significantly different from placebo, although BUP/SAM did improve the overall depressive symptoms severity. Treatment-related AE were mild or moderate in severity	Phase III, NCT02158546
Fava et al. (2020)	ALKS-5461 as adjunctive therapy to ongoing antidepressant, *N* = 385, and 407 MDD patients, respectively, 5 + 6 weeks (two stages)	One of these trials achieved its primary outcome (MADRS total score and Bech-6 score change from baseline to week 6), while the other did not. The pooled analysis of these two trials demonstrated a greater reduction in MADRS total scores from baseline for 2/2 mg BUP/SAM *vs.* placebo from baseline at multiple time points, including the last visit, and a significant average change from baseline to week 3 to the end of study. The overall tolerability was good	Phase III, NCT02158533 Phase III, NCT02218008
NLM (2014b)	ALKS-5461 two titration doses adjunctive to ongoing treatment, *N* = 66 MDD patients with inadequate response to treatment, 8 weeks	No SAE were recorded in either group of titration, while 67.65% of the subjects who received 1-week titration and 87.5% of those with 2-week titration had AE during the study	Phase III, NCT02085135
Thase et al. (2019)	ALKS-5461 as adjunctive therapy, open-label, *N* = 1486 MDD patients unresponsive to prior antidepressant therapy, 52 weeks	AE were reported by 75.7% of the patients, but the majority were of mild or moderate intensity. Discontinuation due to AE was recorded in 10.4% of the cases. SAE were reported in 3.2% of the patients. Improvements in MADRS scores were maintained until the last visit, suggesting durability of antidepressant effect in patients receiving continuous treatment	Phase III, NCT02141399
NLM (2017d)	ALKS-5461 *vs*. placebo adjunctive to current treatment, *N* = 278 TRD patients, 5 + 6 weeks (two stages)	Non-significant differences between groups were reported according to the main outcome measure, MADRS total score. The overall tolerability was good, with no SAE recorded in either stage of this trial	Phase IIIb, NCT03188185

AE, adverse events; DBRCT, double-blind, randomized controlled trial; DXMQ, dextromethorphan hydrobromide and quinidine; HAMD, Hamilton Depression Rating Scale; MADRS, Montgomery Asberg Depression Rating Scale; MDD, major depressive disorder; PGI-I, Patient Global Impression of Improvement; QIDS-SR, Quick Inventory of Depressive Symptomatology-Self-Report; SAE, severe adverse events; TRD, treatment-resistant MDD.

### Cholinergic Receptor Modulators

Pharmacological interventions targeting nicotinic receptors have been explored in multiple psychiatric disorders, for example, MDD, neurocognitive disorders, nicotine use disorder, or schizophrenia (Davidson et al., 2021). **JNJ-39393406** is an investigational product with properties of α7 nicotinic receptors selective positive allosteric modulator, and it can lower agonist and nicotine threshold for activation of these receptors 10–20-fold while increasing the maximum agonist response 17–20-fold (Davidson et al., 2021). In a randomized, double-blind, placebo-controlled, add-on to psychotropics, parallel-group trial, 71 patients diagnosed with MDD were monitored for 2 weeks (Davidson et al., 2021). The primary outcome measures were the Brief Assessment of Cognition in Schizophrenia (BACS) composite score and the MADRS scores (Davidson et al., 2021). No significant difference for the primary outcomes was detected at the end of the study, nor for the secondary outcomes (Davidson et al., 2021). The overall tolerability of JNJ-39393406 was good, without differences in the adverse events rate between active drug and placebo groups (Davidson et al., 2021).

**Ropanicant (SUVN-911)** is a potent α4β2 receptor ligand with oral bioavailability, good brain penetration, and marked antidepressant activity in animal models of depression (Nirogi et al., 2020). A phase I, single-center, open-label, single-dose study evaluated the effect of food, gender, and age on the safety and pharmacokinetic profile of SUVN-911, administered orally in healthy subjects (*N* = 28 participants), but results are not available (NLM, NCT03551288). Another phase I, double-blind, placebo-controlled, single-center clinical study explored the safety, tolerability, and pharmacokinetic profile of single and multiple doses of orally administered SUVN-911 or placebo to healthy male subjects (*N* = 64), but no results are available for this study either (NLM, NCT03155503).

**Scopolamine** is a competitive inhibitor of post-ganglionic muscarinic receptors for acetylcholine, and it acts as a nonselective muscarinic antagonist (Zhang et al., 2017). The effects of scopolamine hydrobromide administration (4 μg/kg i.v.) were evaluated in two trials, a double-blind, placebo-controlled, dose-finding study followed by a double-blind, placebo-controlled, crossover clinical trial (Furey and Drevets, 2006). Adult outpatients diagnosed with MDD or bipolar disorder (*N* = 19) received multiple sessions of i.v. infusions of placebo or scopolamine hydrobromide, and these sessions were 3–5 days apart (Furey and Drevets, 2006). Patients who received a placebo followed by scopolamine showed no significant change in the main outcomes (MADRS and HAMA scores) during the placebo phase, but significant reductions in both depression and anxiety rating scores were observed after scopolamine administration (Furey and Drevets, 2006). Patients who received scopolamine first and placebo second also showed significant reductions in depression and anxiety rating scale scores after scopolamine i.v., relative to baseline, and these effects persisted during the placebo phase (Furey and Drevets, 2006).

Outpatients with MDD (*N* = 23) were enrolled in a double-blind, placebo-controlled, crossover trial, and they were randomized into either a placebo-scopolamine or a scopolamine-placebo sequence (Drevets and Furey, 2010). Scopolamine was administered in 4 μg/kg i.v dose, in repeated sessions, 3–5 days apart (Drevets and Furey, 2010). MADRS scores decreased by 32% in patients who received first scopolamine (*p* < 0.001) *versus* those who received the placebo first, and improvement was significant at the first evaluation that followed scopolamine administration (Drevets and Furey, 2010). Scopolamine administration was associated with drowsiness, blurred vision, dry mouth, lightheadedness, and reduced blood pressure, and no participant dropped out due to side effects (Drevets and Furey, 2010).

In a double-blind, randomized, controlled, phase IV trial, 14 MDD participants received either scopolamine 0.15 mg b.i.d and naltrexone 1 mg b.i.d for 4 weeks or placebo, and they were monitored for 4 weeks (NLM, NCT03386448). According to the results posted by the sponsor, the change of MADRS scores from baseline to the end of the study visit (the primary outcome) was significant in favor of the scopolamine and naltrexone group (*p* = 0.03), and the rate of adverse events in the active group was 25% (mainly nausea) *versus* 0% in the placebo group (NLM, NCT03386448).

A randomized, double-blind, placebo-controlled, phase II trial focused on the evaluation of i.v. scopolamine in patients with bipolar disorder who experience a depressive episode of at least moderate severity is ongoing as of April 2022 (NLM, NCT04211961). The main outcome is the change in the HAMD score at 2 weeks, and the recruitment target is 50 participants (NLM, NCT04211961).

A randomized, controlled trial had the objective of comparing the effects of ketamine + placebo, scopolamine + placebo, and ketamine + scopolamine in patients with treatment-resistant MDD, monitored for up to 4 months, but this study was withdrawn due to lack of funding (NLM, NCT01613820).

A randomized, placebo-controlled, phase II study evaluated the efficacy of i.v. scopolamine (4 μg/kg) in seven patients diagnosed with MDD who are receiving electroconvulsive therapy (ECT) (NLM, NCT01312844). According to the unpublished results posted on clinicaltrials.gov, scopolamine was not significantly superior to placebo in any of the primary outcomes: the change in the HAMD-17 scores was −17.5 *versus* −14.0 (scopolamine *vs*. placebo) at the time of ECT completion (about 2 weeks), the mean time to response for patients receiving ECT was 8.33 *vs*. 5.0 days, and the mean number of ECT sessions to achieve response/remission were 2.33 *versus* 2.5/10 *versus* 6.5 (scopolamine *vs*. placebo) (NLM, NCT01312844).

In another double-blind, placebo-controlled, phase IV clinical trial, 66 adult outpatients with severe MDD were randomized on 1) scopolamine 0.3 mg/ml i.m. in the morning and placebo i.m. in the afternoon, 2) scopolamine 0.3 mg/ml i.m. twice daily, or 3) placebo i.m. (0.9% saline) twice daily (NLM, NCT03131050). All patients also received 10 mg/day of escitalopram for 4 weeks of treatment (NLM, NCT03131050). No results of this trial have been yet released.

In conclusion, targeting cholinergic neurotransmission as a key mechanism for antidepressant activity is supported by several trials, but most of the research on this pharmacodynamic mechanism is still in its early phase (Table 4).

**TABLE 4:** Cholinergic receptor modulators and other agents with antidepressant properties in the pipeline.

Authors	Methodology	Results	Clinical trial phase, trial identifier (if available)
Cholinergic agents			
Davidson et al. (2021)	JNJ-39393406 *vs.* placebo as add-on, DBRCT, *N* = 71 MDD patients, 2 weeks	No significant differences between groups in BACS composite score or MADRS total score were reported at week 2. The overall tolerability was good	Phase II, NCT02677207
NLM (2018h)	Ropanicant (SUVN-911), open-label, single-dose study, *N* = 28 healthy subjects	Primary outcome measure is AUC. Results have not been yet disclosed	Phase I, NCT03551288
NLM (2017e)	SUVN-911 *vs.* placebo, single/multiple doses, DBRCT, *N* = 64 healthy male subjects	Primary outcome measures-ECG, vital signs, C-SSRS. Results have not yet been disclosed	Phase I, NCT03155503
Furey and Drevets (2006)	Scopolamine 4 μg/kg i.v. *vs.* placebo, two DBRCTs, *N* = 19 MDD/BD patients, repeated sessions 3–5 days apart	Patients who received placebo followed by scopolamine showed no significant change in the main outcomes (MADRS and HAMA scores) during the placebo phase, but significant reductions in both outcomes were observed after scopolamine administration. Patients who received scopolamine first and placebo second also showed significant reductions in depression and anxiety rating scale scores after scopolamine i.v., relative to baseline, and these effects persisted during the placebo phase	Phase N/A
Drevets and Furey (2010)	Scopolamine 4 μg/kg i.v. *vs.* placebo, DBRCT, *N* = 23 MDD outpatients, repeated sessions 3–5 days apart	MADRS scores decreased by 32% in patients who received first scopolamine (*p* < 0.001) *vs*. those who received the placebo first, and improvement was significant at the first evaluation that followed scopolamine administration. Scopolamine administration was associated with drowsiness, blurred vision, dry mouth, lightheadedness, and reduced blood pressure, and no participant dropped out due to side effects	Phase II, NCT00369915
NLM (2017f)	Scopolamine 0.15 mg b.i.d. + naltrexone 1 mg b.i.d. *vs.* placebo, DBRCT, *N* = 14 MDD patients, 4 weeks	Unpublished results support a significant change of the MADRS scores from baseline to the end of the study visit (the primary outcome) in favor of the scopolamine and naltrexone group (*p* = 0.03), and the rate of adverse events in the active group was 25% (mainly nausea) *vs*. 0% in the placebo group	Phase IV, NCT03386448
NLM (2019c)	Scopolamine i.v. *vs.* placebo, DBRCT, *N* = 50 (recruitment target), 2 weeks	The main outcome is the change in HAMD score at 2 weeks. The trial is ongoing	Phase II, NCT04211961
NLM (2012d)	Ketamine + placebo, scopolamine + placebo, and ketamine + scopolamine, DBRCT, *N* = 0 MDD participants, 4 months	This study was withdrawn due to a lack of funding	Phase N/A, NCT01613820
NLM (2011a)	Scopolamine 4 μg/kg i.v. *vs.* placebo + ECT, DBRCT, *N* = 7 MDD patients, 2 weeks	Unpublished results suggest that scopolamine was not significantly superior to placebo in any of the primary outcomes: the change in the HAMD-17 scores was −17.5 *vs*. −14.0 (scopolamine *vs*. placebo) at the time of ECT completion (about 2 weeks); the meantime for response for patients receiving ECT was 8.33 *vs*. 5.0 days, and the mean number of ECT sessions to achieve response/remission was 2.33 *vs*. 2.5/10 *vs*. 6.5 (scopolamine *vs*. placebo)	Phase II, NCT01312844
NLM (2017g)	Scopolamine 0.3 mg/ml or 0.6 mg/ml i.m. *vs.* placebo + escitalopram 10 mg/day, DBRCT, N = 66 outpatients with severe MDD, 4 weeks	No results of this trial have been yet released	Phase IV, NCT03131050
Other agents			
NLM (2019d)	MAP4343 *vs.* placebo, DBRCT, *N* = 110 (estimated), TRD patients, 42 days	The primary outcome measure is HAMD’s total score change. The results of this trial have not yet been disclosed	Phase II, NCT03870776
Frye et al. (2000)	Gabapentin *vs.* lamotrigine *vs.* placebo, *N* = 31 treatment-resistant MDD and BD patients, 6 weeks	Response rates (based on CGI ratings of much or very much improved) were 26% for gabapentin, 52% for lamotrigine, and 23% for placebo. The overall tolerability of gabapentin was good	Phase N/A
Arnold et al. (2015)	Pregabalin *vs.* placebo + SSRI/SNRI, cross-over DBRCT, *N* = 197 fibromyalgia + depression patients, periods of 2 × 6 week	Pregabalin significantly improved HADS score, both anxiety and depression scale scores, Fibromyalgia Impact Questionnaire total score, but not EuroQoL 5-dimensions scores	Phase N/A
Timmers et al. (2018)	JNJ-54175446 *vs.* placebo, SAD, N = 77 healthy participants	AE were reported in 56% of the participants, and the most frequently reported was headache (18.6%)	Phase I, NCT02475148
NLM (2019d)	JNJ-54175446 *vs.* placebo, DBRCT, *N* = 142 (estimated), MDD patients with incomplete response to antidepressants, 8 weeks	The primary outcome measure is MADRS total score change. The trial is ongoing	Phase II, NCT04116606
Brin et al. (2020)	OnabotulinumtoxinA (onabotA) 30 U/50U *vs.* placebo, DBRCT, *N* = 255 MDD patients, 24 weeks	Onabot 30U approached significance *vs*. placebo, according to the MADRS scores change, and reached significance at weeks 3 and 9, with secondary endpoints also reaching significance at several time points. The overall tolerability was good	Phase II, NCT02116361
NLM (2011b)	OnabotA (29–40 U) *vs.* placebo, DBRCT, *N* = 30 MDD patients, 6 weeks	Patients were monitored using HAMD-21, and the change in the active drug followed by placebo group was significant *vs*. placebo followed by active drug (−12.7 *vs*. −0.4) at 12 weeks (*p* < 0.001). No SAE were recorded in either group	Phase II, NCT01392963
Finzi and Rosenthal (2014)	OnabotA (29U/40U), *vs.* placebo, DBRCT, *N* = 85 MDD patients, 6 weeks	Response rates (based on MADRS scores) at 6 weeks from the injection date were 52% and 15% in the onabotA *vs*. placebo groups (*p* < 0.001). The remission rate (also based on MADRS score) was 27% *vs*. 7% in the onabotA *vs*. placebo	Phase IV, NCT01556971
NLM (2018i)	OnabotA *vs.* placebo, DBRCT, *N* = 58 (estimated), TRD patients, 6 weeks	The main outcome measure is the proportion of patients with improvement of depressive symptoms based on the MADRS scale at 6 weeks after injection. The trial is ongoing	Phase N/A, NCT03484754
NLM (2009b)	OnabotA (20–50 U), open-label, *N* = 50, MDD and non-depressed individuals, 12 weeks	BDI score change was −14.9 in the MDD group at week 12 *vs*. −2.7 in the healthy volunteers, while the self-esteem improved by three points on RSES *vs*. −0.4 in the healthy participants at the endpoint. The quality of life (WHOQOL-BREF) improved in the MDD group with 0.5 points at week 12 compared to baseline, and 0.2 points in the comparator group	Phase IV, NCT01004042
Monti et al. (2019)	PH-10 low-dose/high-dose *vs.* placebo, DBRCT, *N* = 30 MDD patients, 9 weeks	HAMD-17 total score change at endpoint *vs*. baseline showed a trend for difference (*p* = 0.07), with a greater reduction of depression severity scores for high dose PH10 *vs*. placebo. The positive effects of PH10 were recorded from week 1 for the high dose (*p* = 0.03). No SAE were reported, and the overall tolerability was good	Phase IIa
Binneman et al. (2008)	CP-316,311 *vs.* placebo *vs.* sertraline, DBRCT, *N* = 167 recurrent MDD patients, 6 weeks	The change from baseline in the HAMD score at the final visit was not significantly different between the investigational product group and placebo group, although sertraline did differentiate itself from the placebo	Phase II, NCT00143091
Zobel et al. (2000)	R121919, open-label, *N* = 24 MDD patients, 30 days	The drug was safe and well-tolerated within the 30-day observation period. It induced reductions in depression and anxiety scores using clinician-rated and patient-scored instruments (HAMD, BDI, HAMA, CGI, STAI)	Phase IIa

AE, adverse events; BD, bipolar depression; BDI, Beck Depression Inventory; CGI, Clinical Global Impression; DBRCT, double-blind, randomized controlled trial; ECT, electroconvulsive therapy; HADS, Hospital Anxiety and Depression Scale; HAMA, Hamilton Anxiety Rating Scale; HAMD, Hamilton Depression Rating Scale; MADRS, Montgomery Asberg Depression Rating Scale; MDD, major depressive disorder; PGI-I, Patient Global Impression of Improvement; QIDS-SR, Quick Inventory of Depressive Symptomatology-Self-Report; RSES, Rosenberg Self-Esteem Scale; SAD, single ascending dose; SAE, severe adverse events; SNRI, serotonin and norepinephrine reuptake inhibitor; SSRI, selective serotonin reuptake inhibitors; STAI, State-Trait Anxiety Inventory; TRD, treatment-resistant MDD; WHOQOL-BREF, World Health Organization Quality of Life-BREF.

### Other Agents

Animal studies suggest a possible role of microtubule-associated protein type-2 (MAP-2) as a target for the treatment of depressive disorders due to the association of this pathology with neuronal abnormalities in brain microtubule function, including changes in α-tubulin isoforms (Bianchi and Baulieu, 2012). The synthetic pregnenolone-derivative **MAP4343 (3β-methoxy-pregnenolone)** binds to MAP-2 *in vitro* and increases its ability to stimulate tubulin assembly (Bianchi and Baulieu, 2012). The effects of a single injection of MAP4343 and fluoxetine in naïve Sprague Dawley rats were compared, with positive results for the investigational product (Bianchi and Baulieu, 2012). The MAP4343 had efficacy in the rat forced swimming test, the most widely used model of depression, by decreasing immobility behavior (Bianchi and Baulieu, 2012). These antidepressant effects were accompanied by modifications of α-tubulin isoforms in the hippocampus, amygdala, and prefrontal cortex (Bianchi and Baulieu, 2012). A phase II, double-blind, randomized, placebo-controlled, parallel, multicentric trial was planned to enroll 110 adult patients with TRD (NLM, NCT03870776).

**Gabapentinoids** modulate α2δ subunits of voltage-gated calcium channels and have been explored in numerous psychiatric disorders, MDD included (Vasiliu et al., 2017). A double-blind, randomized, crossover, placebo-controlled study compared the efficacy of gabapentin and lamotrigine monotherapy *versus* placebo in 31 patients with refractory unipolar or bipolar mood disorders who were monitored for 6 weeks (Frye et al., 2000). Response rates (based on CGI ratings of much or very much improved) were 26% for gabapentin, 52% for lamotrigine, and 23% for placebo, with a Cochran’s Q *post hoc* difference between gabapentin and placebo of 0.08 (*p* = 0.70) (Frye et al., 2000). The overall tolerability of gabapentin was good (Frye et al., 2000).

A randomized, placebo-controlled, double-blind, 2-period, 2-way crossover study was composed of two 6-week treatment periods, separated by 2 weeks of taper/wash-out phases, and recruited 197 patients diagnosed with fibromyalgia taking a stable dose of SSRI or serotonin and norepinephrine reuptake inhibitor (SNRI) for comorbid depression (Arnold et al., 2015). These patients were randomized on pregabalin/placebo or placebo/pregabalin (300–450 mg/day) as an adjuvant to the current antidepressant treatment (Arnold et al., 2015). Pregabalin significantly improved Hospital Anxiety Depression Scale (HADS) score, both anxiety and depression scales scores, and Fibromyalgia Impact Questionnaire total score but not EuroQoL 5-dimensions scores (Arnold et al., 2015).

Microglial cells within the central nervous system are presumedly involved in the neuroinflammation that has been associated with multiple neuropsychiatric disorders (Bhattacharya and Ceusters, 2020). Neuroinflammatory drug targets on microglia cells within the central nervous system have been of interest, especially in the last decades. **JNJ-54175446** is a brain penetrant-P2X7 antagonist agent which targets an ATP-activated ion channel, abundantly expressed on microglia and peripheral immune cells (Bhattacharya and Ceusters, 2020). In a first-in-human, placebo-controlled, single ascending dose study, JNJ-54175446 demonstrated in healthy participants (*N* = 77) dose-dependent increases in plasma exposure, cerebrospinal fluid exposure, and *ex vivo* inhibition of IL-1β from human blood (Timmers et al., 2018). Adverse events were reported in 56% of the participants, of which the most frequently reported was headache (18.6%) (Timmers et al., 2018). A phase II, randomized, placebo-controlled, double-blind trial of the antidepressant efficacy of JNJ-54165446 in patients with MDD and incomplete response to monoaminergic antidepressants is currently ongoing, and as the main outcome, it has measured the change in the MADRS score from baseline to week 8 (NLM., NCT04116606).

Another approach to MDD treatment involves nonsystemic options, with the main benefit of avoiding the onset of adverse events and potentially dangerous pharmacokinetic interactions frequently associated with orally administered classical antidepressants. Local injections of **onabotulinumtoxinA (onabotA, BOTOX)** may determine muscle relaxation through a complex process involving the cleaving of SNAP-25 (synaptosomal-associated protein-25 kD) (Brin et al., 2020). The consequence of this process is a lack of neurotransmitter content released from the vesicles in the synaptic cleft, including acetylcholine from motor neurons (Brin et al., 2020).

A 24-week multicenter, randomized, double-blind, placebo-controlled, two-cohort, parallel-group, phase II clinical trial evaluated the effects of 30 and 50 U onabotA in outpatients female patients (*N* = 255) with MDD (Brin et al., 2020). The investigational product or placebo was divided into six or eight glabellar injections (Brin et al., 2020). Onabot 30U approached significance *versus* placebo, according to the MADRS scores change, and reached significance at weeks 3 and 9, with secondary endpoints also reaching significance at several time points (Brin et al., 2020). Onabot 50U did not separate at week 6 from placebo in any variables (Brin et al., 2020). The overall tolerability was good, and the most commonly reported adverse events ( 5% in either of the active treatment groups) were headache, upper respiratory infections, and eyelid ptosis (Brin et al., 2020). OnabotA 30U administered in a single injection during a unique session had a consistent efficacy signal across multiple depression symptom scales for at least 12 weeks (Brin et al., 2020).

In a phase II, randomized, double-blind, cross-over trial, 30 patients diagnosed with MDD received an injection of clostridium botulinum toxin type A neurotoxin complex (29–40 U total injection) in the glabella region or placebo, and after 3 months, they received a placebo injection or Botox to the same region (NLM, NCT01392963). Patients were monitored using HAMD-21, and the change in the active drug followed by placebo group was significant *versus* placebo followed by active drug (−12.7 *vs*. −0.4) at 12 weeks (*p* < 0.001) (NLM, NCT01392963). No SAE were recorded in either group (NLM, NCT01392963).

In a phase IV, randomized trial, a single dose of onambotulinumtoxinA (29 U for females or 40 U for males) or placebo injections was administered into corrugator and procerus frown muscles in 85 patients diagnosed with MDD (Finzi and Rosenthal, 2014). Response rates (MADRS scores decreased by≥50%) at 6 weeks from the injection date were 52% and 15% in the onabotA *versus* placebo groups (*p* < 0.001) (Finzi and Rosenthal, 2014). The remission rate (MADRS score < 10) was 27% *versus* 7% in the onabotA *versus* placebo (Finzi and Rosenthal, 2014).

Another ongoing trial is investigating the effect of onabotA injection in the corrugator and procerus muscle in patients with TRD in comparison to the infiltration in the crow’s feet area, in addition to the current antidepressant treatment (NLM, NCT03484754). The estimated enrollment is 58 participants, and the main outcome measure is the proportion of patients with improvement of depressive symptoms based on the MADRS scale at 6 weeks after injection (NLM, NCT03484754).

Quality of life, depressive symptoms, and self-esteem were the main outcomes in a phase IV, non-randomized, open-label trial, which enrolled 50 patients diagnosed with MDD *versus* non-depressed subjects who received onabotA injections of 20–40 U in five points of the glabellar area (NLM, NCT01004042). The change in BDI scores was −14.9 in the MDD group at week 12 *versus* −2.7 in the healthy volunteers, while the self-esteem improved by three points on the Rosenberg Self-Esteem Scale *versus* −0.4 in the healthy participants at endpoint (NLM, NCT01004042). The quality of life (WHOQOL-BREF score) improved in the MDD group with 0.5 points at week 12 compared to baseline and 0.2 points in the comparator group (NLM, NCT01004042).

**PH-10** is a synthetic, odorless neuroactive steroid from the family of pherines, formulated for intranasal administration (Monti et al., 2019). It engages nasal chemosensory receptors and modulates neural circuits in the limbic amygdala and other basal forebrain structures, inducing antidepressant-like effects (Monti et al., 2019). In a single site exploratory phase IIa study, 30 patients with MDD were randomized to 8 weeks of self-administered intranasal PH10 low dose (3.2 μg), high dose (6.4 μg), or placebo (Monti et al., 2019). The analysis of HAMD-17 changes at endpoint *versus* baseline showed a trend for difference (*p* = 0.07), with a greater reduction of depression severity scores for high dose PH10 *versus* placebo (Monti et al., 2019). The positive effects of PH10 were recorded starting from week 1 for the high dose (*p* = 0.03) (Monti et al., 2019). No serious adverse effects were reported, and the overall tolerability was good (Monti et al., 2019).

Dysregulation of the hypothalamic-pituitary-adrenal (HPA) axis has been suggested as an important pathogenetic mechanism in MDD, but up to date, there are no drugs targeting this system approved for use in patients with mood disorders (Menke, 2019). **CRH-1 receptor antagonists** have been investigated in patients with MDD or anxiety disorders without significant results, but several authors consider that a more homogenous group of participants in clinical trials (i.e., those with significant CRH signaling dysfunction) may help detect a signal for these molecules (Menke, 2019). A phase II, randomized, parallel assignment, 6-week, fixed-dose, double-blind, double-dummy, placebo and sertraline controlled, multicenter trial evaluated the safety and efficacy of CP-316,311 (a selective CRH-1 antagonist) in outpatients with recurrent MDD (*N* = 167 participants) (Binneman et al., 2008). The efficacy of 400 mg of CP-316,311 twice daily was compared with 100 mg sertraline daily or placebo, and the interim analysis led to the trial termination (Binneman et al., 2008). The change from baseline in the HAMD score at the final visit was not significantly different between the investigational product group and placebo group, although sertraline did differentiate itself from the placebo (Binneman et al., 2008).

Another high-affinity, CRH-1 receptor antagonist, R121919, was investigated in a clinical trial (*N* = 24 patients with MDD), and it was safe and well-tolerated within the 30-day observation period (Zobel et al., 2000). The CRH-1 blockade did not impair the baseline corticotropin/cortisol activity, and it did not have such an effect following an exogenous CRH challenge (Zobel et al., 2000). R121919 induced reductions in depression and anxiety scores using clinician-rated and patient-scored instruments (Zobel et al., 2000).

In conclusion, these molecules with distinct pharmacodynamic properties indicate, besides the complexity of the MDD pathogenesis, the need for further exploration of different central and peripheric ways (e.g., steroid hormones, ion channel modulators, or locally administered exoproteins) to decrease the severity of depressive symptoms (Table 4).

**FIGURE 5:**
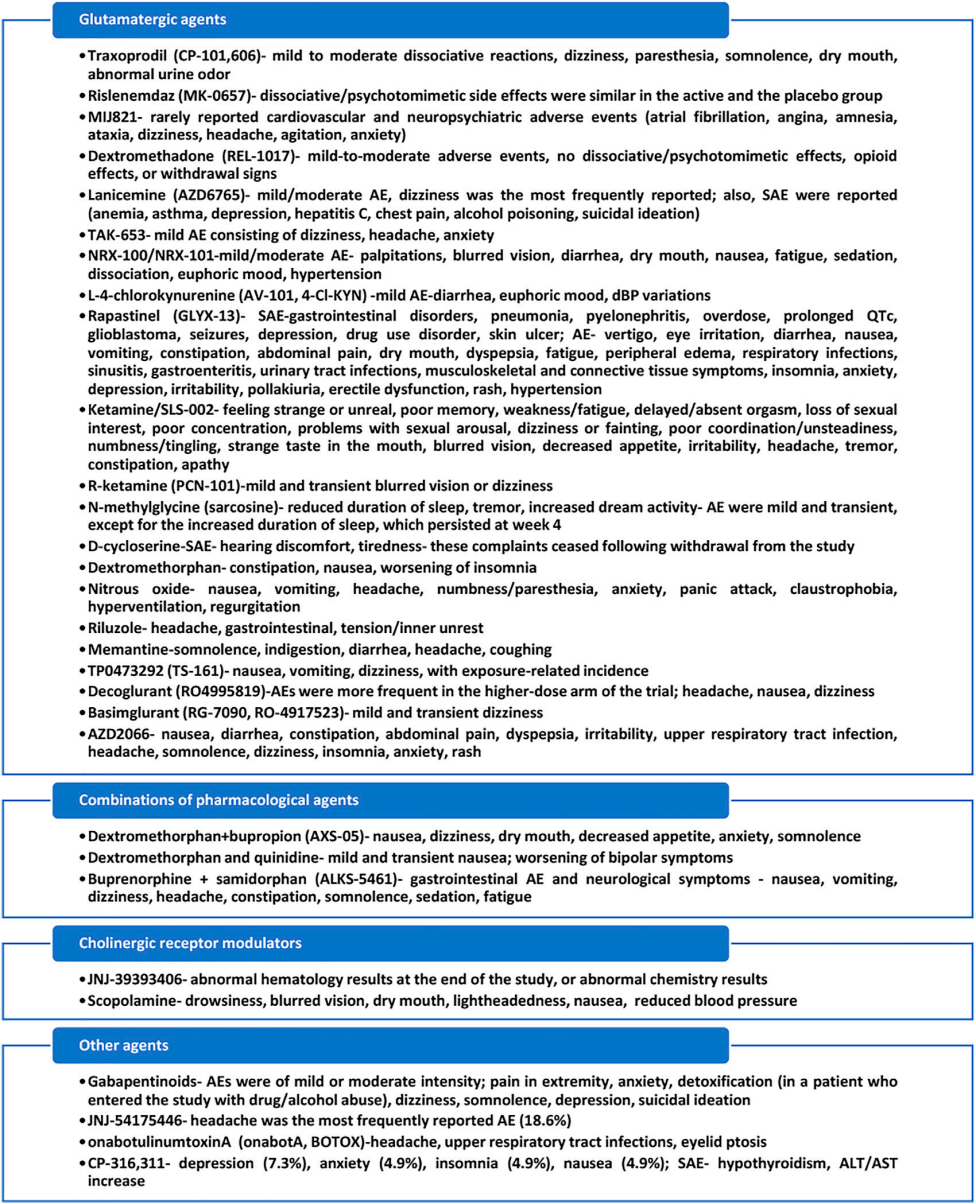
Main adverse events identified in clinical trials exploring new antidepressant agents. AE, adverse events; dBP, diastolic blood pressure; SAE, severe adverse events. Based on data from Preskorn et al. (2008); Ibrahim et al. (2012); Ghaemi et al. (2021); Fava et al. (2022); Sanacora et al. (2017); O’Donnell et al. (2021); NLM, NCT02974010; Park et al. (2020); NLM, NCT02192099; Lapidus et al. (2014); Leal et al. (2021); Huang et al. (2013); Heresco-Levy et al. (2013); Majeed et al. (2021); Nagele et al. (2015); Zarate et al. (2004); NLM, NCT00305578; Umbricht et al. (2020); Quiroz et al. (2016); NLM, NCT01145755; Axsome Therapeuticals (2019); Axsome Therapeuticals (2020); Kelly and Lieberman (2014); Fava et al. (2016); NLM, NCT03188185; Davidson et al. (2021); Drevets and Furey (2010); NLM, NCT03386448; Arnold et al. (2015); Timmers et al. (2018); Brin et al. (2020); Binneman et al. (2008).

A synthesis of the safety and tolerability data available for investigational products reviewed in this study is presented in Figure 5.

## Conclusion

Multiple non-monoaminergic pathways are considered of interest in clinical research in the treatment of depressive disorders. Glutamatergic agents are by far the most extensively researched, and several sub-categories have been identified: antagonists of GluN2B subunits of NMDA receptors (seven investigational products), NMDA-receptor antagonists (14 agents), AMPA receptor potentiators (two agents), and metabotropic receptor antagonists (four agents). These agents were investigated in phases I–III of clinical trials. One sestrin modulator is investigated in phases I and II of clinical trials. Combinations of pharmacological agents (i.e., AXS-05, DXMQ, AVP-786, and ALKS-5461) are investigated for their antidepressant properties in phases II and III of clinical trials. Two cholinergic agents (i.e., JNJ-39393406 and SUVN-911) are explored in phases I and Ii of clinical trials, but phase Ii trials have undisclosed results. Other agents (i.e., MAP4343, gabapentin, pregabalin, JNJ-54175446, onabotA, PH10, CP-316, 311, and R1219191) have been studied in phases I–IV clinical trials. In conclusion, there is an abundance of investigational products that reached phases II and III of clinical research, although it is too early to formulate a prognosis if any of these agents will be approved any time soon for MDD or bipolar depression.

Limitations of this review refer to the possible exclusion of antidepressants in the pipeline due to the pre-formulated criteria of this search and the lack of availability of relevant data regarding the current status of investigation for these products in the explored references.

## Data Availability

The original contributions presented in the study are included in the article/Supplementary Material. Further inquiries can be directed to the corresponding author.

## References

[B1] Agbo F. Bui K. H. Zhou D. (2017). Population Pharmacokinetic Analysis of Lanicemine (AZD6765), an NMDA Channel Blocker, in Healthy Subjects and Patients with Major Depressive Disorder. J. Clin. Pharm. Ther. 42 (5), 539–546. 10.1111/jcpt.12541 28474366

[B2] Alexopoulos G. S. (2019). Mechanisms and Treatment of Late-Life Depression. Transl. Psychiatry 9, 188. 10.1038/s41398-019-0514-6 31383842 PMC6683149

[B3] Arnold L. M. Sarzi-Puttini P. Arsenault P. Khan T. Bhadra Brown P. Clair A. (2015). Efficacy and Safety of Pregabalin in Patients with Fibromyalgia and Comorbid Depression Taking Concurrent Antidepressant Medication: A Randomized, Placebo-Controlled Study. J. Rheumatol. 42 (7), 1237–1244. 10.3899/jrheum.141196 26034150

[B4] Arsova A. Møller T. C. Vedel L. Hansen J. L. Foster S. R. Gregory K. J. (2020). Detailed In Vitro Pharmacological Characterization of Clinically Tested Negative Allosteric Modulators of the Metabotropic Glutamate Receptor 5. Mol. Pharmacol. 98 (1), 49–60. 10.1124/mol.119.119032 32358164 PMC7705108

[B5] Axsome Therapeutics (2019). ASCEND Phase 2 Trial of AXS-05 in MDD Topline Results. Available at: https://axsometherapeuticsinc.gcs-web.com/static-files/229c52a8-ab22-4dd3-b36f-08904c22cd4b (Accessed January 28, 2022).

[B6] Axsome Therapeutics (2021). Axsome Therapeutics Announces AXS-05 Achieves Primary and Key Secondary Endpoints in the MERIT Phase 2 Trial in Treatment Resistant Depression. Available at: https://www.globenewswire.com/news-release/2021/08/09/2276951/33090/en/Axsome-Therapeutics-Announces-AXS-05-Achieves-Primary-and-Key-Secondary-Endpoints-in-the-MERIT-Phase-2-Trial-in-Treatment-Resistant-Depression.html (Accessed January 28, 2022).

[B7] Axsome Therapeutics (2020). Axsome Therapeutics Presents New Data from GEMINI Phase 3 Trial with AXS-05 Demonstrating Rapid and Significant Improvements in Patient-Reported Outcomes in Major Depressive Disorder. Available at: https://www.globenewswire.com/news-release/2020/09/14/2092779/33090/en/Axsome-Therapeutics-Presents-New-Data-from-GEMINI-Phase-3-Trial-with-AXS-05-Demonstrating-Rapid-and-Significant-Improvements-in-Patient-Reported-Outcomes-in-Major-Depressive-Disord.html (Accessed January 28, 2022).

[B8] Bahji A. Vazquez G. H. Zarate C. A. Jr. (2021). Comparative Efficacy of Racemic Ketamine and Esketamine for Depression: A Systematic Review and Meta-Analysis. J. Affect Disord. 278, 542–555. 10.1016/j.jad.2020.09.071 33022440 PMC7704936

[B9] Bahr R. Lopez A. Rey J. A. (2019). Intranasal Esketamine (SpravatoTM) for Use in Treatment-Resistant Depression in Conjunction with an Oral Antidepressant. P T 44 (6), 340-342, 344-346, 375. 31160868 PMC6534172

[B10] Bhattacharya A. Ceusters M. (2020). Targeting Neuroinflammation with Brain Penetrant P2X7 Antagonists as Novel Therapeutics for Neuropsychiatric Disorders. Neuropsychopharmacology 45, 234–235. 10.1038/s41386-019-0502-9 31477815 PMC6879571

[B11] Bianchi M. Baulieu E. E. (2012). 3β-Methoxy-pregnenolone (MAP4343) as an Innovative Therapeutic Approach for Depressive Disorders. Proc. Natl. Acad. Sci. U. S. A. 109 (5), 1713–1718. 10.1073/pnas.1121485109 22307636 PMC3277154

[B12] Binneman B. Feltner D. Kolluri S. Shi Y. Qiu R. Stiger T. (2008). A 6-week Randomized, Placebo-Controlled Trial of CP-316,311 (A Selective CRH1 Antagonist) in the Treatment of Major Depression. Am. J. Psychiatry 165 (5), 617–620. 10.1176/appi.ajp.2008.07071199 18413705

[B13] Biospace (2020). Axsome Therapeutics Announces Topline Results of the STRIDE-1 Phase 3 Trial in Treatment Resistant Depression and Expert Call to Discuss Clinical Implications. Available at: https://www.biospace.com/article/releases/axsome-therapeutics-announces-topline-results-of-the-stride-1-phase-3-trial-in-treatment-resistant-depression-and-expert-call-to-discuss-clinical-implications/ (Accessed January 28, 2022).

[B14] Brennan B. P. Hudson J. I. Jensen J. E. McCarthy J. Roberts J. L. Prescot A. P. (2010). Rapid Enhancement of Glutamatergic Neurotransmission in Bipolar Depression Following Treatment with Riluzole. Neuropsychopharmacology 35 (3), 834–846. 10.1038/npp.2009.191 19956089 PMC3055603

[B15] Brin M. F. Durgam S. Lum A. James L. Liu J. Thase M. E. (2020). OnabotulinumtoxinA for the Treatment of Major Depressive Disorder: a Phase 2 Randomized, Double-Blind, Placebo-Controlled Trial in Adult Females. Int. Clin. Psychopharmacol. 35 (1), 19–28. 10.1097/YIC.0000000000000290 31609787 PMC6903360

[B16] Brooks M. (2015). Rapid-acting Antidepressants Show Promise. Available at: https://www.medscape.com/viewarticle/838794 (Accessed February 5, 2022).

[B17] Burgdorf J. Kroes R. A. Zhang X. L. Gross A. L. Schmidt M. Weiss C. (2015a). Rapastinel (GLYX-13) Has Therapeutic Potential for the Treatment of Post-traumatic Stress Disorder: Characterization of a NMDA Receptor-Mediated Metaplasticity Process in the Medial Prefrontal Cortex of Rats. Behav. Brain Res. 294, 177–185. 10.1016/j.bbr.2015.07.039 26210936 PMC4702501

[B18] Burgdorf J. Zhang X. L. Weiss C. Gross A. Boikess S. R. Kroes R. A. (2015b). The Long-Lasting Antidepressant Effects of Rapastinel (GLYX-13) Are Associated with a Metaplasticity Process in the Medial Prefrontal Cortex and hippocampus. Neuroscience 308, 202–211. 10.1016/j.neuroscience.2015.09.004 26343295 PMC4721228

[B19] Callahan R. J. Au J. D. Paul M. Liu C. Yost C. S. (2004). Functional Inhibition by Methadone of N-Methyl-D-Aspartate Receptors Expressed in Xenopus Oocytes: Stereospecific and Subunit Effects. Anesth. Analg. 98 (3), 653–contents. 10.1213/01.ane.0000099723.75548.df 14980914

[B20] Chen K. T. Wu C. H. Tsai M. H. Wu Y. C. Jou M. J. Huang C. C. (2017). Antidepressant-like Effects of Long-Term Sarcosine Treatment in Rats with or without Chronic Unpredictable Stress. Behav. Brain Res. 316, 1–10. 10.1016/j.bbr.2016.06.004 27555541

[B21] Chen M. H. Cheng C. M. Gueorguieva R. Lin W. C. Li C. T. Hong C. J. (2019). Maintenance of Antidepressant and Antisuicidal Effects by D-Cycloserine Among Patients with Treatment-Resistant Depression Who Responded to Low-Dose Ketamine Infusion: a Double-Blind Randomized Placebo-Control Study. Neuropsychopharmacology 44 (12), 2112–2118. 10.1038/s41386-019-0480-y 31421635 PMC6898334

[B22] Chen S. L. Lee S. Y. Chang Y. H. Chen P. S. Lee I. H. Wang T. Y. (2014). Therapeutic Effects of Add-On Low-Dose Dextromethorphan Plus Valproic Acid in Bipolar Disorder. Eur. Neuropsychopharmacol. 24 (11), 1753–1759. 10.1016/j.euroneuro.2014.09.001 25262178

[B23] Conley A. A. Norwood A. E. Q. Hatvany T. C. Griffith J. D. Barber K. E. (2021). Efficacy of Ketamine for Major Depressive Episodes at 2, 4, and 6-weeks Post-treatment: A Meta-Analysis. Psychopharmacol. Berl. 238 (7), 1737–1752. 10.1007/s00213-021-05825-8 33787963

[B24] Davidson M. Levi L. Park J. Nastas I. Ford L. Rassnick S. (2021). The Effects of JNJ-39393406 a Positive Allosteric Nicotine Modulator on Mood and Cognition in Patients with Unipolar Depression: A Double-Blind, Add-On, Placebo-Controlled Trial. Eur. Neuropsychopharmacol. 51, 33–42. 10.1016/j.euroneuro.2021.04.020 34023797

[B25] Doble A. (1996). The Pharmacology and Mechanism of Action of Riluzole. Neurology 47 (6 Suppl. 4), S233–S241. 10.1212/wnl.47.6_suppl_4.233s 8959995

[B26] Drevets W. C. Furey M. L. (2010). Replication of Scopolamine's Antidepressant Efficacy in Major Depressive Disorder: a Randomized, Placebo-Controlled Clinical Trial. Biol. Psychiatry 67 (5), 432–438. 10.1016/j.biopsych.2009.11.021 20074703 PMC3264395

[B27] Fava M. Memisoglu A. Thase M. E. Bodkin J. A. Trivedi M. H. de Somer M. (2016). Opioid Modulation with Buprenorphine/samidorphan as Adjunctive Treatment for Inadequate Response to Antidepressants: A Randomized Double-Blind Placebo-Controlled Trial. Am. J. Psychiatry 173 (5), 499–508. 10.1176/appi.ajp.2015.15070921 26869247

[B28] Fava M. Stahl S. Pani L. De Martin S. Pappagallo M. Guidetti C. (2022). REL-1017 (Esmethadone) as Adjunctive Treatment in Patients with Major Depressive Disorder: A Phase 2a Randomized Double-Blind Trial. Am. J. Psychiatry 179 (2), 122–131. 10.1176/appi.ajp.2021.21020197 34933568

[B29] Fava M. Thase M. E. Trivedi M. H. Ehrich E. Martin W. F. Memisoglu A. (2020). Opioid System Modulation with Buprenorphine/samidorphan Combination for Major Depressive Disorder: Two Randomized Controlled Studies. Mol. Psychiatry 25 (7), 1580–1591. 10.1038/s41380-018-0284-1 30374191 PMC7303008

[B30] Finzi E. Rosenthal N. E. (2014). Treatment of Depression with onabotulinumtoxinA: a Randomized, Double-Blind, Placebo-Controlled Trial. J. Psychiatr. Res. 52, 1–6. 10.1016/j.jpsychires.2013.11.006 24345483

[B31] Fogaça M. V. Fukumoto K. Franklin T. Liu R. J. Duman C. H. Vitolo O. V. (2019). N-Methyl-D-aspartate Receptor Antagonist D-Methadone Produces Rapid, mTORC1-dependent Antidepressant Effects. Neuropsychopharmacology 44 (13), 2230–2238. 10.1038/s41386-019-0501-x 31454827 PMC6898593

[B32] Fried E. I. Nesse R. M. (2014). The Impact of Individual Depressive Symptoms on Impairment of Psychosocial Functioning. PLoS One 9 (2), e90311. 10.1371/journal.pone.0090311 24587318 PMC3938686

[B33] Frye M. A. Ketter T. A. Kimbrell T. A. Dunn R. T. Speer A. M. Osuch E. A. (2000). A Placebo-Controlled Study of Lamotrigine and Gabapentin Monotherapy in Refractory Mood Disorders. J. Clin. Psychopharmacol. 20 (6), 607–614. 10.1097/00004714-200012000-00004 11106131

[B34] Furey M. L. Drevets W. C. (2006). Antidepressant Efficacy of the Antimuscarinic Drug Scopolamine: A Randomized, Placebo-Controlled Clinical Trial. Arch. Gen. Psychiatry 63 (10), 1121–1129. 10.1001/archpsyc.63.10.1121 17015814 PMC3250308

[B35] Gant T. G. (2014). Using Deuterium in Drug Discovery: Leaving the Label in the Drug. J. Med. Chem. 57 (9), 3595–3611. 10.1021/jm4007998 24294889

[B36] Ghaemi N. Sverdlov A. Shelton R. Litman R. (2021). Efficacy and Safety of MIJ821 in Patients with Treatment-Resistant Depression: Results from a Randomized, Placebo-Controlled, Proof-Of-Concept Study. Eur. Psychiatr. 64 (S1), S334–S335. 10.1192/j.eurpsy.2021.897

[B37] Hara H. Suzuki A. Kunugi A. Tajima Y. Yamada R. Kimura H. (2021). TAK-653, an AMPA Receptor Potentiator with Minimal Agonistic Activity, Produces an Antidepressant-like Effect with a Favorable Safety Profile in Rats. Pharmacol. Biochem. Behav. 211, 173289. 10.1016/j.pbb.2021.173289 34655652

[B38] Hecking J. Davoudian P. A. Wilkinson S. T. (2021). Emerging Therapeutics Based on the Amino Acid Neurotransmitter System: An Update on the Pharmaceutical Pipeline for Mood Disorders. Chronic Stress (Thousand Oaks) 5, 24705470211020446. 10.1177/24705470211020446 34124495 PMC8175843

[B39] Heerlein K. De Giorgi S. Degraeve G. Frodl T. Hagedoorn W. Oliveira-Maia A. J. (2022). Real-world Evidence from a European Cohort Study of Patients with Treatment Resistant Depression: Healthcare Resource Utilization. J. Affect Disord. 298 (Pt), 442–450. 10.1016/j.jad.2021.11.004 34742998

[B40] Heresco-Levy U. Gelfin G. Bloch B. Levin R. Edelman S. Javitt D. C. (2013). A Randomized Add-On Trial of High-Dose D-Cycloserine for Treatment-Resistant Depression. Int. J. Neuropsychopharmacol. 16 (3), 501–506. 10.1017/S1461145712000910 23174090

[B41] Heresco-Levy U. Javitt D. C. Gelfin Y. Gorelik E. Bar M. Blanaru M. (2006). Controlled Trial of D-Cycloserine Adjuvant Therapy for Treatment-Resistant Major Depressive Disorder. J. Affect Disord. 93 (1), 239–243. 10.1016/j.jad.2006.03.004 16677714

[B42] Huang C. C. Wei I. H. Huang C. L. Chen K. T. Tsai M. H. Tsai P. (2013). Inhibition of glycine Transporter-I as a Novel Mechanism for the Treatment of Depression. Biol. Psychiatry 74 (10), 734–741. 10.1016/j.biopsych.2013.02.020 23562005

[B43] Ibrahim L. Diaz Granados N. Jolkovsky L. Brutsche N. Luckenbaugh D. A. Herring W. J. (2012). A Randomized, Placebo-Controlled, Crossover Pilot Trial of the Oral Selective NR2B Antagonist MK-0657 in Patients with Treatment-Resistant Major Depressive Disorder. J. Clin. Psychopharmacol. 32 (4), 551–557. 10.1097/JCP.0b013e31825d70d6 22722512 PMC3438886

[B44] Ignácio Z. M. Réus G. Z. Arent C. O. Abelaira H. M. Pitcher M. R. Quevedo J. (2016). New Perspectives on the Involvement of mTOR in Depression as Well as in the Action of Antidepressant Drugs. Br. J. Clin. Pharmacol. 82 (5), 1280–1290. 10.1111/bcp.12845 26613210 PMC5061805

[B45] Johnson J. W. Kotermanski S. E. (2006). Mechanism of Action of Memantine. Curr. Opin. Pharmacol. 6 (1), 61–67. 10.1016/j.coph.2005.09.007 16368266

[B46] Kalmoe M. C. Janski A. M. Zorumski C. F. Nagele P. Palanca B. J. Conway C. R. (2020). Ketamine and Nitrous Oxide: The Evolution of NMDA Receptor Antagonists as Antidepressant Agents. J. Neurol. Sci. 412, 116778. 10.1016/j.jns.2020.116778 32240970

[B47] Kato T. Pothula S. Liu R. J. Duman C. H. Terwilliger R. Vlasuk G. P. (2019). Sestrin Modulator NV-5138 Produces Rapid Antidepressant Effects via Direct mTORC1 Activation. J. Clin. Invest 129 (6), 2542–2554. 10.1172/JCI126859 30990795 PMC6546461

[B48] Kelly T. F. Lieberman D. Z. (2014). The Utility of the Combination of Dextromethorphan and Quinidine in the Treatment of Bipolar II and Bipolar NOS. J. Affect Disord. 167, 333–335. 10.1016/j.jad.2014.05.050 25016490

[B49] Kessler R. C. Berglund P. Demler O. Jin R. Koretz D. Merikangas K. R. (2003). The Epidemiology of Major Depressive Disorder: Results from the National Comorbidity Survey Replication (NCS-R). JAMA 289 (23), 3095–3105. 10.1001/jama.289.23.3095 12813115

[B50] Kishimoto T. Chawla J. M. Hagi K. Zarate C. A. Jr Kane J. M. Bauer M. (2016). Single-dose Infusion Ketamine and Non-ketamine N-Methyl-D-Aspartate Receptor Antagonists for Unipolar and Bipolar Depression: a Meta-Analysis of Efficacy, Safety and Time Trajectories. Psychol. Med. 46 (7), 1459–1472. 10.1017/S0033291716000064 26867988 PMC5116384

[B51] Lacerda A. L. T. (2020). Esketamine/ketamine for Treatment-Resistant Depression. Braz J. Psychiatry 42 (6), 579–580. 10.1590/1516-4446-2020-0996 32401866 PMC7678896

[B52] Lapidus K. A. B. Levitch C. F. Perez A. M. Brallier J. W. Parides M. K. Soleimani L. (2014). A Randomized Controlled Trial of Intranasal Ketamine in Major Depressive Disorder. Biol. Psychiatry 76 (12), 970–976. 10.1016/j.biopsych.2014.03.026 24821196 PMC4185009

[B53] Leal G. C. Bandeira I. D. Correia-Melo F. S. Telles M. Mello R. P. Vieira F. (2021). Intravenous Arketamine for Treatment-Resistant Depression: Open-Label Pilot Study. Eur. Arch. Psychiatry Clin. Neurosci. 271 (3), 577–582. 10.1007/s00406-020-01110-5 32078034

[B54] Levy B. Manive E. (2012). Functional Outcome in Bipolar Disorder: The Big Picture. Depress. Res. Treat. 2012, 949248. 10.1155/2012/949248. 21961062 10.1155/2012/949248 PMC3180778

[B55] Lindemann L. Porter R. H. Scharf S. H. Kuennecke B. Bruns A. von Kienlin M. (2015). Pharmacology of Basimglurant (RO4917523, RG7090), a Unique Metabotropic Glutamate Receptor 5 Negative Allosteric Modulator in Clinical Development for Depression. J. Pharmacol. Exp. Ther. 353 (1), 213–233. 10.1124/jpet.114.222463 25665805

[B56] Majeed A. Xiong J. Teopiz K. M. Ng J. Ho R. Rosenblat J. D. (2021). Efficacy of Dextromethorphan for the Treatment of Depression: a Systematic Review of Preclinical and Clinical Trials. Expert Opin. Emerg. Drugs 26 (1), 63–74. 10.1080/14728214.2021.1898588 33682569

[B57] Mathew S. J. Murrough J. W. aan het Rot M. Collins K. A. Reich D. L. Charney D. S. (2010). Riluzole for Relapse Prevention Following Intravenous Ketamine in Treatment-Resistant Depression: A Pilot Randomized, Placebo-Controlled Continuation Trial. Int. J. Neuropsychopharmacol. 13 (1), 71–82. 10.1017/S1461145709000169 19288975 PMC3883127

[B58] Menke A. (2019). Is the HPA axis as Target for Depression Outdated, or Is There a New Hope? Front. Psychiatry 10, 101. 10.3389/fpsyt.2019.00101 30890970 PMC6413696

[B59] Moher D. Liberati A. Tetzlaff J. Altman D. G. (2009). Preferred Reporting Items for Systematic Reviews and Meta-Analyses: the PRISMA Statement. BMJ 339 (4), b2535–269. 10.1371/journal.pmed.1000097 19622551 PMC2714657

[B60] Moher D. Shamseer L. Clarke M. Ghersi D. Liberati A. Petticrew M. (2015). Preferred Reporting Items for Systematic Review and Meta-Analysis Protocols (PRISMA-P) 2015 Statement. Syst. Rev. 4 (1), 1. 10.1186/2046-4053-4-1 25554246 PMC4320440

[B61] Monti L. Nicolini H. Liebowitz M. R. Hanover R. (2019). A Placebo Controlled Trial of PH10: Test for a New Rapidly Acting Intranasally Administered Antidepressant. Br. J. Pharm. Med. Res. 4 (6), 2157–2168. 10.24942/bjpmr.2019.604(

[B62] Moskal J. R. Burch R. Burgdorf J. S. Kroes R. A. Stanton P. K. Disterhoft J. F. (2014). GLYX-13, an NMDA Receptor glycine Site Functional Partial Agonist Enhances Cognition and Produces Antidepressant Effects without the Psychotomimetic Side Effects of NMDA Receptor Antagonists. Expert Opin. Investig. Drugs 23 (2), 243–254. 10.1517/13543784.2014.852536 PMC4721220 24251380

[B63] Murphy N. Ramakrishnan N. Vo-Le B. Vo-Le B. Smith M. A. Iqbal T. (2021). A Randomized Cross-Over Trial to Define Neurophysiological Correlates of AV-101 N-Methyl-D-Aspartate Receptor Blockade in Healthy Veterans. Neuropsychopharmacology 46 (4), 820–827. 10.1038/s41386-020-00917-z 33318635 PMC8027791

[B64] Murrough J. W. Wade E. Sayed S. Ahle G. Kiraly D. D. Welch A. (2017). Dextromethorphan/quinidine Pharmacotherapy in Patients with Treatment Resistant Depression: A Proof of Concept Clinical Trial. J. Affect Disord. 218, 277–283. 10.1016/j.jad.2017.04.072 28478356

[B65] Nagele P. Duma A. Kopec M. Gebara M. A. Parsoei A. Walker M. (2015). Nitrous Oxide for Treatment-Resistant Major Depression: A Proof-Of-Concept Trial. Biol. Psychiatry 78 (1), 10–18. 10.1016/j.biopsych.2014.11.016 25577164

[B66] National Library of Medicine U.S (2010a). 6-week Study Treatment to Evaluate the Safety and Effectiveness of AZD2006 in Patients with Major Depressive Disorder. Available at: https://clinicaltrials.gov/ct2/show/NCT01145755 (Accessed February 3, 2022).

[B67] National Library of Medicine U.S (2006a). A Double-Blind Placebo-Controlled Trial of Riluzole in Bipolar Depression. Available at: https://clinicaltrials.gov/ct2/show/results/NCT00376220 (Accessed April 4, 2022).

[B68] National Library of Medicine U.S (2017a). A Study of ALKS-5461 for the Treatment Refractory Major Depressive Disorder (MDD). Available at: https://clinicaltrials.gov/ct2/show/results/NCT03188185 (Accessed February 4, 2022).

[B69] National Library of Medicine U.S (2014a). A Study of Different Titration Schedules of ALKS-5461 in Adults with Major Depressive Disorder (MDD). Available at: https://clinicaltrials.gov/ct2/show/results/NCT02085135 (Accessed February 4, 2022).

[B70] National Library of Medicine U.S (2015a). A Study of Safety, Tolerability, and Pharmacokinetics of Multiple-Ascending Dose Basimglurant in Healthy Subjects and in Patients with Major Depressive Disorder (MDD). Available at: https://clinicaltrials.gov/ct2/show/NCT02433093 (Accessed January 30, 2022).

[B71] National Library of Medicine U.S (2020). A Study of SLS-002 (Intranasal Racemic Ketamine) in Adults with Major Depressive Disorder at Imminent Risk of Suicide. Available at: https://clinicaltrials.gov/ct2/show/NCT04669665 (Accessed February 5, 2022).

[B72] National Library of Medicine U.S (2011a). A Study of the Use of IV Scopolamine to Augment the Efficacy of Electroconvulsive Therapy (ECT). Available at: https://clinicaltrials.gov/ct2/show/NCT01312844 (Accessed April 2, 2022).

[B73] National Library of Medicine U.S (2021a). A Study to Assess the Efficacy and Safety of REL-1017 as Adjunctive Treatment for Major Depressive Disorder (MDD) (RELIANCE-I). Available at: https://clinicaltrials.gov/ct2/show/NCT04688164 (Accessed February 3, 2022).

[B74] National Library of Medicine U.S. (2021b). A Study to Assess the Efficacy and Safety of REL-1017 as Adjunctive Treatment for Major Depressive Disorder (MDD) (RELIANCE-II).Available at: https://clinicaltrials.gov/ct2/show/NCT04855747 . Accessed February 3, 2022.

[B75] National Library of Medicine U.S (2021c). A Study to Assess the Efficacy and Safety of REL-1017 as Monotherapy for Major Depressive Disorder (MDD) (RELIANCE-III). Available at: https://clinicaltrials.gov/ct2/show/NCT05081167 (Accessed February 3, 2022).

[B76] National Library of Medicine U.S (2017b). A Study to Investigate the Safety, Tolerability and Pharmacokinetics of SUVN-911 in Healthy Subjects (SUVN-911). Available at: https://clinicaltrials.gov/ct2/show/NCT03155503 (Accessed February 5, 2022).

[B77] National Library of Medicine U.S (2018b). AGN-241751 in the Treatment of Major Depressive Disorder. Available at: https://clinicaltrials.gov/ct2/show/NCT03726658 (Accessed January 30, 2022).

[B78] National Library of Medicine U.S (2021d). Antidepressant Effects of TS-161 in Treatment-Resistant Depression. Available at: https://clinicaltrials.gov/ct2/show/NCT04821271 (Accessed February 3, 2022).

[B79] National Library of Medicine U.S (2019a). Antidepressant Trial with P2X7 Antagonist JNJ-54175446. Available at: https://clinicaltrials.gov/ct2/show/NCT04116606 (Accessed February 5, 2022).

[B80] National Library of Medicine U.S (2002). Clinical Trial of Memantine for Major Depression. Available at: https://clinicaltrials.gov/ct2/show/NCT00040261 (Accessed April 4, 2022).

[B81] National Library of Medicine U.S (2018c). Effect of Food, Gender, and Age on the Pharmacokinetic Profile of SUVN-911 in Healthy Subjects (SUVN-911). Available at: https://clinicaltrials.gov/ct2/show/NCT03551288 (Accessed February 5, 2022).

[B82] National Library of Medicine U.S (2017c). Effectiveness Study of Scopolamine Combined with Escitalopram in Patients with MDD (SCE). Available at: https://clinicaltrials.gov/ct2/show/NCT03131050 (Accessed April 2, 2022).

[B83] National Library of Medicine U.S (2012b). Efficacy and Safety of GLYX-13 in Subjects with Inadequate/partial Response to Antidepressants. Available at: https://clinicaltrials.gov/ct2/show/NCT01684163 (Accessed January 30, 2022).

[B84] National Library of Medicine U.S (2017d). Efficacy and Safety of TAK-653 in Treatment-Resistant Depression. Available at: https://clinicaltrials.gov/ct2/show/NCT03312894 (Accessed February 5, 2022).

[B85] National Library of Medicine U.S (2010b). Efficacy and Tolerability of Riluzole in Treatment-Resistant Depression. Available at: https://clinicaltrials.gov/ct2/show/results/NCT01204918 (Accessed April 4, 2022).

[B86] National Library of Medicine U.S (2014b). Efficacy, Safety, and Tolerability of AVP-786 as an Adjunctive Therapy in Patients with Major Depressive Disorder with an Inadequate Response to Antidepressant Treatment. Available at: https://clinicaltrials.gov/ct2/show/NCT02153502 (Accessed February 3, 2022).

[B87] National Library of Medicine U.S (2021e). Evaluation of Efficacy and Safety of Add-On Sarcosine in Patients with Major Depressive Disorder. Available at: https://clinicaltrials.gov/ct2/show/NCT04975100 (Accessed February 1, 2022).

[B88] National Library of Medicine U.S (2018d). Improving Therapeutic Learning in Depression: Proof of Concept. Available at: https://clinicaltrials.gov/ct2/show/results/NCT02376257 (Accessed February 2, 2022).

[B89] National Library of Medicine U.S (2018e). Infiltration of Onabotulinum Toxin A in Resistant Depression: Comparison of Two Facial Injection Sites. (OnaDEP). Available at: https://clinicaltrials.gov/ct2/show/NCT03484754 (Accessed February 6, 2022).

[B90] National Library of Medicine U.S (2017e). Inhaled Nitrous Oxide for Treatment-Resistant Depression: Optimizing Dosing Strategies (NARSAD). Available at: https://clinicaltrials.gov/ct2/show/study/NCT03283670 (Accessed February 9, 2022).

[B91] National Library of Medicine U.S (2012c). Investigate Efficacy and Safety of RO4995819 vs. Placebo as Adjunct Tx in Patients with Major Depressive Disorder. Available at: https://clinicaltrials.gov/ct2/show/NCT01733654 (Accessed February 3, 2022).

[B92] National Library of Medicine U.S (2012a). Ketamine and Scopolamine Infusions for Treatment-Resistant Major Depressive Disorder. Available at: https://clinicaltrials.gov/ct2/show/NCT01613820 (Accessed April 2, 2022).

[B93] National Library of Medicine U.S (2015b). Ketamine for Treatment Resistant Late-Life Depression. Available at: https://clinicaltrials.gov/ct2/show/NCT02556606 (Accessed February 19, 2022).

[B94] National Library of Medicine U.S (2017f). Low-dose Ketamine and Postpartum Depression in Parturients with Prenatal Depression. Available at: https://clinicaltrials.gov/ct2/show/NCT03336541 (Accessed February 19, 2022).

[B95] National Library of Medicine U.S (2006c). Memantine Augmentation of Antidepressants. Available at: https://clinicaltrials.gov/ct2/show/NCT00344682 (Accessed April 4, 2022).

[B96] National Library of Medicine U.S (2006b). Memantine Augmentation of Lamotrigine Incomplete-Response in Bipolar Depression. Available at: https://clinicaltrials.gov/ct2/show/NCT00305578 (Accessed April 4, 2022).

[B97] National Library of Medicine U.S (2009a). N-methylglycine (Sarcosine) Treatment for Depression. Available at: https://clinicaltrials.gov/ct2/show/NCT00977353 (Accessed February 1, 2022).

[B98] National Library of Medicine U.S (2018f). NRX 101 for Moderate Bipolar Depression and Suicidal Ideation (MBD). Availableat: https://clinicaltrials.gov/ct2/show/NCT03395392 (Accessed January 30, 2022).

[B99] National Library of Medicine U.S (2018g). NRX 101 Glx Biomarker Validation Study (NRX-GLX). Available at: https://clinicaltrials.gov/ct2/show/NCT03402152 (Accessed January 30, 2022).

[B100] National Library of Medicine U.S (2018h). NRX-101 for Maintenance of Remission from Severe Bipolar Depression in Patients with Suicidal Ideation (SBD-ASIB). Available at: https://clinicaltrials.gov/ct2/show/NCT03396068 (Accessed January 30, 2022).

[B101] National Library of Medicine U.S (2018a). NRX100 vs. Placebo for Rapid Stabilization of Acute Suicidal Ideation and Behavior in Bipolar Depression (Severe BD). Available at: https://clinicaltrials.gov/ct2/show/NCT03396601 (Accessed January 30, 2022).

[B102] National Library of Medicine U.S (2019b). Open Label Extension for GLYX-13-C-202, NCT01684163. Available at: https://clinicaltrials.gov/ct2/show/results/NCT02192099 (Accessed January 30, 2022).

[B103] National Library of Medicine U.S (2021f). Open Label Study to Assess the Safety of REL-1017 for Major Depressive Disorder (RELIANCE-OS). Available at: https://clinicaltrials.gov/ct2/show/NCT04855760 (Accessed February 3, 2022).

[B104] National Library of Medicine U.S (2021g). Phase 1 Evaluation of (2R,6R)-Hydroxynorketamine. Available at: https://clinicaltrials.gov/ct2/show/NCT04711005 (Accessed January 31, 2022).

[B105] National Library of Medicine U.S (2021h). Phase 2 Study of NV-5138 in Adults with Treatment Resistant Depression. Available at: https://clinicaltrials.gov/ct2/show/NCT05066672 (Accessed February 3, 2022).

[B106] National Library of Medicine U.S (2009b). Quality of Life and Self-Esteem after Botox® Injections in Depressed and Non-depressed Patients. Available at: https://clinicaltrials.gov/ct2/show/results/NCT01004042 (Accessed February 6, 2022).

[B107] National Library of Medicine U.S (2019c). RESIST: Administration of MAP4343 in Antidepressant Non-responders Patients Experiencing a Major Depressive Episode. Available at: https://clinicaltrials.gov/ct2/show/NCT03870776 (Accessed February 5, 2022).

[B108] National Library of Medicine U.S (2012d). Riluzole Augmentation Pilot in Depression (RAPID) Trial (RAPID). Available at: https://clinicaltrials.gov/ct2/show/study/NCT01703039 (Accessed April 3, 2022).

[B109] National Library of Medicine U.S (2003). Riluzole to Treat Depression in Bipolar Disorder. Available at: https://clinicaltrials.gov/ct2/show/study/NCT00054704 (Accessed April 4, 2022).

[B110] National Library of Medicine U.S (2001). Riluzole to Treat Major Depression. Available at: https://clinicaltrials.gov/ct2/show/record/NCT00026052 (Accessed April 3, 2022).

[B111] National Library of Medicine U.S (2010c). Safety and Efficacy of EVT-101 in Treatment-Resistant Depression. Available at: https://clinicaltrials.gov/ct2/show/NCT01128452 (Accessed January 29, 2022).

[B112] National Library of Medicine U.S (2013). Safety and Pharmacokinetics of NRX-1074 in Normal Volunteers. Available at: https://clinicaltrials.gov/ct2/show/NCT01856556 (Accessed February 18, 2022).

[B113] National Library of Medicine U.S (2018i). Safety, Tolerability, PK and Efficacy of Single Doses of NV-5138 in Healthy Volunteers and Subjects with Treatment-Resistant Depression. Available at: https://clinicaltrials.gov/ct2/show/NCT03606395 (Accessed February 3, 2022).

[B114] National Library of Medicine U.S (2019d). Scopolamine in Bipolar Disorder (SCOPE-BD). Available at: https://clinicaltrials.gov/ct2/show/NCT04211961 (Accessed April 2, 2022).

[B115] National Library of Medicine U.S (2016). Sequential Therapy for the Treatment of Severe Bipolar Depression (STABIL-B). Available at: https://clinicaltrials.gov/ct2/show/results/NCT02974010 (Accessed January 30, 2022).

[B116] National Library of Medicine U.S (2021i). Study of Efficacy and Safety of MIJ821 in Addition to Comprehensive Standard of Care on the Rapid Reduction of Symptoms of Major Depressive Disorder in Subjects Who Have Suicidal Ideation with Intent. Available at: https://clinicaltrials.gov/ct2/show/NCT04722666 (Accessed February 5, 2022).

[B117] National Library of Medicine U.S (2015c). Study of Safety, Tolerability and Pharmacokinetics of NRX-1074 in Normal Healthy Volunteers (NRX-1074). Available at: https://clinicaltrials.gov/ct2/show/NCT02366364 (Accessed online February 18, 2022).

[B118] National Library of Medicine U.S (2015d). TAK-653 Escalating Single and Multiple Dose Study in Healthy Participants. Available at: https://clinicaltrials.gov/ct2/show/results/NCT02561156 (Accessed February 5, 2022).

[B119] National Library of Medicine U.S (2017g). The Safety and Efficacy of Naltrexone and Scopolamine Utilized in the Treatment of Major Depression. Available at: https://clinicaltrials.gov/ct2/show/NCT03386448 (Accessed April 2, 2022).

[B120] National Library of Medicine U.S (2011b). The Treatment of Depression with Botulinum Type A Toxin. Available at: https://clinicaltrials.gov/ct2/show/results/NCT01392963 (Accessed February 6, 2022).

[B121] Nguyen L. Thomas K. L. Lucke-Wold B. P. Cavendish J. Z. Crowe M. S. Matsumoto R. R. (2016). Dextromethorphan: An Update on its Utility for Neurological and Neuropsychiatric Disorders. Pharmacol. Ther. 159, 1–22. 10.1016/j.pharmthera.2016.01.016 26826604

[B122] Nirogi R. Mohammed A. R. Shinde A. K. Ravella S. R. Bogaraju N. Subramanian R. (2020). Discovery and Development of 3-(6-Chloropyridine-3-yloxymethyl)-2-azabicyclo[3.1.0]hexane Hydrochloride (SUVN-911): A Novel, Potent, Selective, and Orally Active Neuronal Nicotinic Acetylcholine α4β2 Receptor Antagonist for the Treatment of Depression. J. Med. Chem. 63 (6), 2833–2853. 10.1021/acs.jmedchem.9b00790 32026697

[B123] O'Donnell P. Dijkstra F. M. Damar U. Quanhong L. de Goede A. A. Xu L. (2021). Transcranial Magnetic Stimulation as a Translational Biomarker for AMPA Receptor Modulation. Transl. Psychiatry 11 (1), 325. 10.1038/s41398-021-01451-2 34045439 PMC8160137

[B124] Park L. T. Kadriu B. Gould T. D. Zanos P. Greenstein D. Evans J. W. (2020). A Randomized Trial of the N-Methyl-D-Aspartate Receptor glycine Site Antagonist Prodrug 4-chlorokynurenine in Treatment-Resistant Depression. Int. J. Neuropsychopharmacol. 23 (7), 417–425. 10.1093/ijnp/pyaa025 32236521 PMC7387765

[B125] Pearlstein T. Howard M. Salisbury A. Zlotnick C. (2009). Postpartum Depression. Am. J. Obstet. Gynecol. 200 (4), 357–364. 10.1016/j.ajog.2008.11.033 19318144 PMC3918890

[B126] Petrescu B. Vasile D. Vasiliu O. Tudor C. Mangalagiu A. Ungureanu D. (2014). P.2.f.008 SSRI Dose Escalation versus Duloxetine in Treatment of Major Depressive Disorder Not Responding to Initial SSRI. Eur. Neuropsychopharmacol. 24 (2), S455–S456. 10.1016/s0924-977x(14)70729-1

[B127] Poleszak E. Stasiuk W. Szopa A. Wyska E. Serefko A. Oniszczuk A. (2016). Traxoprodil, a Selective Antagonist of the NR2B Subunit of the NMDA Receptor, Potentiates the Antidepressant-like Effects of Certain Antidepressant Drugs in the Forced Swim Test in Mice. Metab. Brain Dis. 31, 803–814. 10.1007/s11011-016-9810-5 26924124 PMC4933725

[B128] Pothula S. Liu R. J. Wu M. Sliby A. N. Picciotto M. R. Banerjee P. (2021). Positive Modulation of NMDA Receptors by AGN-241751 Exerts Rapid Antidepressant-like Effects via Excitatory Neurons. Neuropsychopharmacology 46 (4), 799–808. 10.1038/s41386-020-00882-7 33059355 PMC8027594

[B129] Preskorn S. Macaluso M. Mehra D. O. Zammit G. Moskal J. R. Burch R. M. (2015). Randomized Proof of Concept Trial of GLYX-13, an N-Methyl-D-Aspartate Receptor glycine Site Partial Agonist, in Major Depressive Disorder Nonresponsive to a Previous Antidepressant Agent. J. Psychiatr. Pract. 21 (2), 140–149. 10.1097/01.pra.0000462606.17725.93 25782764

[B130] Preskorn S. H. Baker B. Kolluri S. Menniti F. S. Krams M. Landen J. W. (2008). An Innovative Design to Establish Proof of Concept of the Antidepressant Effects of the NR2B Subunit Selective N-Methyl-D-Aspartate Antagonist, CP-101,606, in Patients with Treatment-Refractory Major Depressive Disorder. J. Clin. Psychopharmacol. 28 (6), 631–637. 10.1097/JCP.0b013e31818a6cea 19011431

[B131] PRNewswire (2021b). Perception Neuroscience’s PCN-101 (R-Ketamine) Demonstrates Tolerability in Phase 1 Single Ascending Dose Study. Available at: https://www.prnewswire.com/news-releases/perception-neurosciences-pcn-101-r-ketamine-demonstrates-tolerability-in-phase-1-single-ascending-dose-study-301231491.html (Accessed February 5, 2022).

[B132] PRNewswire (2021a). Seelos Therapeutics Presents a Poster on SLS-002 (Intranasal Racemic Ketamine) at the 2021 IASR/AFSP International Summit on Suicide Research. Available at: https://www.prnewswire.com/news-releases/seelos-therapeutics-presents-a-poster-on-sls-002-intranasal-racemic-ketamine-at-the-2021-iasrafsp-international-summit-on-suicide-research-301407611.html (Accessed February 5, 2022).

[B133] Quiroz J. A. Tamburri P. Deptula D. Banken L. Beyer U. Rabbia M. (2016). Efficacy and Safety of Basimglurant as Adjunctive Therapy for Major Depression: A Randomized Clinical Trial. JAMA Psychiatry 73 (7), 675–684. 10.1001/jamapsychiatry.2016.0838 27304433

[B134] Sakurai H. Yonezawa K. Tani H. Mimura M. Bauer M. Uchida H. (2022). Novel Antidepressants in the Pipeline (Phase II and III): A Systematic Review of the US Clinical Trials Registry. Pharmacopsychiatry. 10.1055/a-1714-9097 PMC9259184 35045580

[B135] Sanacora G. Johnson M. R. Khan A. Atkinson S. D. Riesenberg R. R. Schronen J. P. (2017). Adjunctive Lanicemine (AZD6765) in Patients with Major Depressive Disorder and History of Inadequate Response to Antidepressants: A Randomized, Placebo-Controlled Study. Neuropsychopharmacology 42 (4), 844–853. 10.1038/npp.2016.224 27681442 PMC5312066

[B136] Sanacora G. Smith M. A. Pathak S. Su H. L. Boeijinga P. H. McCarthy D. J. (2014). Lanicemine: a Low-Trapping NMDA Channel Blocker Produces Sustained Antidepressant Efficacy with Minimal Psychotomimetic Adverse Effects. Mol. Psychiatry 19 (9), 978–985. 10.1038/mp.2013.130 24126931 PMC4195977

[B137] Sanacora G. Zarate C. A. Krystal J. H. Manji H. K. (2008). Targeting the Glutamatergic System to Develop Novel, Improved Therapeutics for Mood Disorders. Nat. Rev. Drug Discov. 7 (5), 426–437. 10.1038/nrd2462 18425072 PMC2715836

[B138] Sanacora G. Kendell S. F. Levin Y. Simen A. A. Fenton L. R. Coric V. (2007). Preliminary Evidence of Riluzole Efficacy in Antidepressant-Treated Patients with Residual Depressive Symptoms. Biol. Psychiatry 61 (6), 822–825. 10.1016/j.biopsych.2006.08.037 17141740 PMC2754299

[B139] Sattar Y. Wilson J. Khan A. M. Adnan M. Azzopardi Larios D. Shrestha S. (2018). A Review of the Mechanism of Antagonism of N-Methyl-D-Aspartate Receptor by Ketamine in Treatment-Resistant Depression. Cureus 10 (5), e2652. 10.7759/cureus.2652 30034974 PMC6051558

[B140] Sengupta S. Giaime E. Narayan S. Hahm S. Howell J. O'Neill D. (2019). Discovery of NV-5138, the First Selective Brain mTORC1 Activator. Sci. Rep. 9, 4107. 10.1038/s41598-019-40693-5 30858438 PMC6412019

[B141] Smith E. G. Deligiannidis K. M. Ulbricht C. M. Landolin C. S. Patel J. K. Rothschild A. J. (2013). Antidepressant Augmentation Using the N-Methyl-D-Aspartate Antagonist Memantine: a Randomized, Double-Blind, Placebo-Controlled Trial. J. Clin. Psychiatry 74 (10), 966–973. 10.4088/JCP.12m08252 24229746 PMC4000742

[B142] Solomon D. A. Leon A. C. Coryell W. H. Endicott J. Li C. Fiedorowicz J. G. (2016). Longitudinal Course of Bipolar I Disorder: Duration of Mood Episodes. Arch. Gen. Psychiatry 67 (4), 339–347. 10.1001/archgenpsychiatry.2010.15 PMC3677763 20368510

[B143] Stroebel D. Buhl D. L. Knafels J. D. Chanda P. K. Green M. Sciabola S. (2016). A Novel Binding Mode Reveals Two Distinct Classes of NMDA Receptor GluN2B-Selective Antagonists. Mol. Pharmacol. 89, 541–551. 10.1124/mol.115.103036 26912815 PMC4859819

[B144] Thase M. E. Stanford A. D. Memisoglu A. Martin W. Claxton A. Bodkin J. A. (2019). Results from a Long-Term Open-Label Extension Study of Adjunctive Buprenorphine/samidorphan Combination in Patients with Major Depressive Disorder. Neuropsychopharmacology 44 (13), 2268–2276. 10.1038/s41386-019-0451-3 31254971 PMC6897901

[B145] Timmers M. Ravenstijn P. Xi L. Triana-Baltzer G. Furey M. van Hemelryck S. (2018). Clinical Pharmacokinetics, Pharmacodynamics, Safety, and Tolerability of JNJ-54175446, a Brain Permeable P2X7 Antagonist, in a Randomised Single-Ascending Dose Study in Healthy Participants. J. Psychopharmacol. 32 (12), 1341–1350. 10.1177/0269881118800067 30260294

[B146] Umbricht D. Niggli M. Sanwald-Ducray P. Deptula D. Moore R. Grünbauer W. (2020). Randomized, Double-Blind, Placebo-Controlled Trial of the mGlu2/3 Negative Allosteric Modulator Decoglurant in Partially Refractory Major Depressive Disorder. J. Clin. Psychiatry 81 (4), 18m12470. 10.4088/JCP.18m12470 32663909

[B147] Vasiliu O. Vasile D. Daniel V. Mangalagiu A. G. Petrescu B. M. Tudor C. (2017). Efficacy and Tolerability of Calcium Channel Alpha-2-Delta Ligands in Psychiatric Disorders. Rjmm 120 (2), 27–31. 10.55453/rjmm.2017.120.2.4

[B148] Vasiliu O. Vasile D. (2016). Risk Factors and Quality of Life in Late-Life Depressive Disorders. Romanian J. Mil. Med. CXIX (3), 24–28.

[B149] Wallace M. White A. Grako K. A. Lane R. Cato A. J. Snodgrass H. R. (2017). Randomized, Double-Blind, Placebo-Controlled, Dose-Escalation Study: Investigation of the Safety, Pharmacokinetics, and Antihyperalgesic Activity of L-4-Chlorokynurenine in Healthy Volunteers. Scand. J. Pain 17, 243–251. 10.1016/j.sjpain.2017.05.004 29229209

[B150] Watanabe M. Marcy B. Hiroki A. Watase H. Kinoshita K. Iijima M. (2021). Evaluation of the Safety, Tolerability, and Pharmacokinetic Profiles of TP0473292 (TS-161), A Prodrug of a Novel Orthosteric mGlu2/3 Receptor Antagonist TP0178894, in Healthy Subjects and its Antidepressant-like Effects in Rodents. Int. J. Neuropsychopharmacol. 25 (2), 106–117. 10.1093/ijnp/pyab062 PMC8832229 34534292

[B151] Wilkinson S. T. Sanacora G. (2019). A New Generation of Antidepressants: an Update on the Pharmaceutical Pipeline for Novel and Rapid-Acting Therapeutics in Mood Disorders Based on Glutamate/GABA Neurotransmitter Systems. Drug Discov. Today 24 (2), 606–615. 10.1016/j.drudis.2018.11.007 30447328 PMC6397075

[B152] Yang C. Shirayama Y. Zhang J. C. Ren Q. Yao W. Ma M. (2015). R-ketamine: a Rapid-Onset and Sustained Antidepressant without Psychotomimetic Side Effects. Transl. Psychiatry 5 (9), e632. 10.1038/tp.2015.136 26327690 PMC5068814

[B153] Zajecka J. M. Stanford A. D. Memisoglu A. Martin W. F. Pathak S. (2019). Buprenorphine/samidorphan Combination for the Adjunctive Treatment of Major Depressive Disorder: Results of a Phase III Clinical Trial (FORWARD-3). Neuropsychiatr. Dis. Treat. 15, 795–808. 10.2147/NDT.S199245 31040679 PMC6459143

[B154] Zanos P. Moaddel R. Morris P. J. Georgiou P. Fischell J. Elmer G. I. (2016). NMDAR Inhibition-independent Antidepressant Actions of Ketamine Metabolites. Nature 533 (7604), 481–486. 10.1038/nature17998 27144355 PMC4922311

[B155] Zanos P. Piantadosi S. C. Wu H. Q. Pribut H. J. Dell M. J. Can A. (2015). The Prodrug 4-chlorokynurenine Causes Ketamine-like Antidepressant Effects, but Not Side Effects, by NMDA/GlycineB-site Inhibition. J. Pharmacol. Exp. Ther. 355 (1), 76–85. 10.1124/jpet.115.225664 26265321 PMC4576668

[B156] Zarate C. A. Jr Mathews D. Ibrahim L. Chaves J. F. Marquardt C. Ukoh I. (2013). A Randomized Trial of a Low-Trapping Nonselective N-Methyl-D-Aspartate Channel Blocker in Major Depression. Biol. Psychiatry 74 (4), 257–264. 10.1016/j.biopsych.2012.10.019 23206319 PMC3594049

[B157] Zarate C. A. Jr. Payne J. L. Quiroz J. Sporn J. Denicoff K. K. Luckenbaugh D. (2004). An Open-Label Trial of Riluzole in Patients with Treatment-Resistant Major Depression. Am. J. Psychiatry 161, 171–174. 10.1176/appi.ajp.161.1.171 14702270

[B158] Zarate C. A. Jr. Quiroz J. A. Singh J. B. Denicoff K. D. De Jesus G. Luckenbaugh D. A. (2005). An Open-Label Trial of the Glutamate-Modulating Agent Riluzole in Combination with Lithium for the Treatment of Bipolar Depression. Biol. Psychiatry 57 (4), 430–432. 10.1016/j.biopsych.2004.11.023 15705360

[B159] Zarate C. A. Jr. Singh J. B. Quiroz J. A. De Jesus G. Denicoff K. K. Luckenbaugh D. A. (2006). A Double-Blind, Placebo-Controlled Study of Memantine in the Treatment of Major Depression. Am. J. Psychiatry 163 (1), 153–155. 10.1176/appi.ajp.163.1.153 16390905

[B160] Zhang J. C. Li S. X. Hashimoto K. (2014). R (-)-ketamine Shows Greater Potency and Longer Lasting Antidepressant Effects Than S (+)-ketamine. Pharmacol. Biochem. Behav. 116, 137–141. 10.1016/j.pbb.2013.11.033 24316345

[B161] Zhang X. C. Farrell N. Haronian T. Hack J. (2017). Postoperative Anticholinergic Poisoning: Concealed Complications of a Commonly Used Medication. J. Emerg. Med. 53 (4), 520–523. 10.1016/j.jemermed.2017.05.003 28756934

[B162] Zobel A. W. Nickel T. Künzel H. E. Ackl N. Sonntag A. Ising M. (2000). Effects of the High-Affinity Corticotropin-Releasing Hormone Receptor 1 Antagonist R121919 in Major Depression: the First 20 Patients Treated. J. Psychiatr. Res. 34 (3), 171–181. 10.1016/s0022-3956(00)00016-9 10867111

